# Partial Loss of Ataxin-1 Function Contributes to Transcriptional Dysregulation in Spinocerebellar Ataxia Type 1 Pathogenesis

**DOI:** 10.1371/journal.pgen.1001021

**Published:** 2010-07-08

**Authors:** Juan Crespo-Barreto, John D. Fryer, Chad A. Shaw, Harry T. Orr, Huda Y. Zoghbi

**Affiliations:** 1 Interdepartmental Program in Cell and Molecular Biology, Baylor College of Medicine, Houston, Texas, United States of America; 2 Department of Molecular and Human Genetics, Baylor College of Medicine, Houston, Texas, United States of America; 3 Institute of Human Genetics, Department of Biochemistry, Biophysics, and Molecular Biology, Department of Laboratory Medicine and Pathology, University of Minnesota, Minneapolis, Minnesota, United States of America; 4 Departments of Neuroscience and Pediatrics, Baylor College of Medicine, Houston, Texas, United States of America; 5 Howard Hughes Medical Institute, Baylor College of Medicine, Houston, Texas, United States of America; Stanford University School of Medicine, United States of America

## Abstract

Spinocerebellar ataxia type 1 (SCA1) is a dominantly inherited neurodegenerative disease caused by expansion of a CAG repeat that encodes a polyglutamine tract in ATAXIN1 (ATXN1). Molecular and genetic data indicate that SCA1 is mainly caused by a gain-of-function mechanism. However, deletion of wild-type ATXN1 enhances SCA1 pathogenesis, whereas increased levels of an evolutionarily conserved paralog of ATXN1, Ataxin 1-Like, ameliorate it. These data suggest that a partial loss of ATXN1 function contributes to SCA1. To address this possibility, we set out to determine if the SCA1 disease model (*Atxn1^154Q/+^* mice) and the loss of Atxn1 function model (*Atxn1^−/−^* mice) share molecular changes that could potentially contribute to SCA1 pathogenesis. To identify transcriptional changes that might result from loss of function of ATXN1 in SCA1, we performed gene expression microarray studies on cerebellar RNA from *Atxn1^−/−^* and *Atxn1^154Q/+^* cerebella and uncovered shared gene expression changes. We further show that mild overexpression of *Ataxin-1-Like* rescues several of the molecular and behavioral defects in *Atxn1* ^−/−^ mice. These results support a model in which *Ataxin 1-Like* overexpression represses SCA1 pathogenesis by compensating for a partial loss of function of *Atxn1*. Altogether, these data provide evidence that partial loss of Atxn1 function contributes to SCA1 pathogenesis and raise the possibility that loss-of-function mechanisms contribute to other dominantly inherited neurodegenerative diseases.

## Author Summary

Spinocerebellar Ataxia type 1 (SCA1) is one of nine neurodegenerative diseases caused by an increase in the number of the amino acid glutamine in their respective proteins. Genetic studies have pointed to the fact that the glutamine expansion in Ataxin-1 causes SCA1 by causing Ataxin-1 to gain some function(s). Here, we demonstrate that in addition to the toxic gain-of-function mechanism, partial loss of the normal functions of Ataxin-1 contributes to SCA1. Ataxin-1 forms protein complexes with Capicua, a protein that silences expression of other genes, and we found that in SCA1 mouse models the levels of these complexes are reduced, resulting in increased expression of some genes. We also demonstrate that increased levels of Ataxin-1-Like, a protein that is similar to Ataxin-1 and protects against mutant Ataxin-1 in mice, rescues molecular and behavioral defects in mice deficient in Ataxin-1. These results show that Ataxin-1-Like compensates for loss of Ataxin-1 and that Ataxin-1 and Ataxin-1-Like share some normal functions. Together, these findings suggest that rescue of SCA1 symptoms by Ataxin-1-Like could be partly due to restoration of lost normal functions of Ataxin-1 in mice that express the mutant polyglutamine-expanded Ataxin-1.

## Introduction

Polyglutamine diseases are caused by the expansion of an unstable translated CAG repeats that encode a polyglutamine tract in unrelated proteins [1], [2]. There are nine dominantly inherited neurodegenerative disorders caused by expanded polyglutamine tracts: Huntington's disease (HD), spinobulbar muscular atrophy (SBMA), dentatorubropallidoluysian atrophy (DRPLA), and six spinocerebellar ataxias (SCA1–3, 6,7 and 17) [1]–[7]. Several genetic studies have revealed that loss of the involved proteins in humans and mice does not cause neurodegeneration, leading to the conclusion that the polyglutamine expanded protein causes disease by a dominant gain-of-function mechanism whereby it confers toxic properties to the host proteins [8]–[14]. The importance of protein context and sub-cellular localization has been highlighted in SCA1 pathogenesis because expansion of the polyglutamine tract is necessary but not sufficient to cause neurodegeneration. For example, overexpression of polyglutamine-expanded ATXN1 that has a single serine residue mutated to alanine (S776A) does not lead to Purkinje cell degeneration, and overexpression of polyglutamine-expanded ATXN1 lacking a functional nuclear localization signal or lacking the AXH domain is not toxic in mice [15]–[17]. These data revealed key domains in ATXN1 that are critical for SCA1 pathogenesis, and indicated that mutant ATXN1 must be localized in the nucleus to exert toxicity. Furthermore, these data suggest that perhaps normal interactions or functions of ATXN1 are relevant to SCA1 pathogenesis.

Several protein interactors of ATXN1 have been identified to date. Among these, there are various transcriptional regulators, including the Capicua homolog CIC, SMRTER, HDAC3, GFI-1 and RORα. Some of these factors modify the pathogenesis of SCA1 in mice and fly models [15], [18]–[20]. For instance, *Rorα* haploinsufficiency results in enhanced pathogenesis in SCA1 transgenic mice [20]. Furthermore, SCA1 transgenic mice share common gene expression changes with the *staggerer* mice, which have a spontaneous mutation in the *Rorα* gene that leads to cerebellar defects and ataxia [20]–[22].

Recent evidence shows that altered interactions of ATXN1 with its native partners contribute to SCA1 pathogenesis. Studies in the knock-in mouse model of SCA1, *Atxn1^154Q/+^*, show that polyglutamine-expanded Atxn1 prefers the formation of a protein complex with the RNA splicing factor RBM17 while concomitantly diminishing the formation of Atxn1 complexes with CIC, a transcriptional repressor [23]. These data suggest an endogenous role of ATXN1 in transcriptional regulation that might be altered in SCA1.

Even though much of the genetic evidence suggests that SCA1 is mainly caused by a gain-of-function mechanism, additional data also suggest that partial loss of Atxn1 function might contribute to pathogenesis. We demonstrated that removing the wild-type copy of *Atxn1* in the knock-in mouse model of SCA1 (*Atxn1^154Q/−^*) leads to worsened SCA1 phenotypes [23]. Moreover, phenotypes and neuropathology in *Atxn1^154Q/+^*mice are partially suppressed by the mild overexpression of an evolutionarily conserved paralog, *Ataxin-1-Like* (*Atxn1L*) [19], [24]. Atxn1L shares high homology with Atxn1, however it lacks the polyglutamine tract, and it interacts with all tested proteins that bind to Atxn1 [19], [24], [25]. Together, these studies indicate that altering the levels of wild-type Atxn1 and its paralog Atxn1L results in strong modulation of SCA1 phenotypes.

Taken together, these data led us to hypothesize that SCA1 pathogenesis results from a gain-of-function mechanism, with partial loss of Atxn1 function potentially contributing to the disease. Since Cic protein levels are also reduced in the *Atxn1* ^−/−^ mouse model [18], we predict that loss of functional Atxn1-Cic complexes could lead to common transcriptional defects in *Atxn1* ^−/−^ and *Atxn1^154Q/+^* mice. Given the predicted role of Atxn1 in transcriptional regulation, we set out to test this possibility by performing microarray analyses on *Atxn1* ^−/−^ and *Atxn1^154Q/+^*cerebella. We show that *Atxn1* ^−/−^ and *Atxn1^154Q/+^*mice share many common transcriptional and molecular phenotypes, some potentially involving alterations in Atxn1-Cic-mediated transcriptional repression. Loss of *Atxn1* function also results in several cerebellar transcriptional changes in common with mice lacking *Rorα*, another transcription factor that genetically and physically interacts with ATXN1. Finally, we demonstrate that overexpression of the *Atxn1*-related gene *Atxn1L* can rescue some of the molecular and behavioral defects caused by loss-of-function of *Atxn1*, strongly suggesting that *Atxn1L*-mediated suppression of SCA1 neuropathology could be due to restoration of the partial loss of Atxn1 function component in the disease.

## Results

### *Atxn1* ^−/−^ mice share cerebellar transcriptional alterations with *Atxn1^154Q/+^*mice

**Figure 1:**
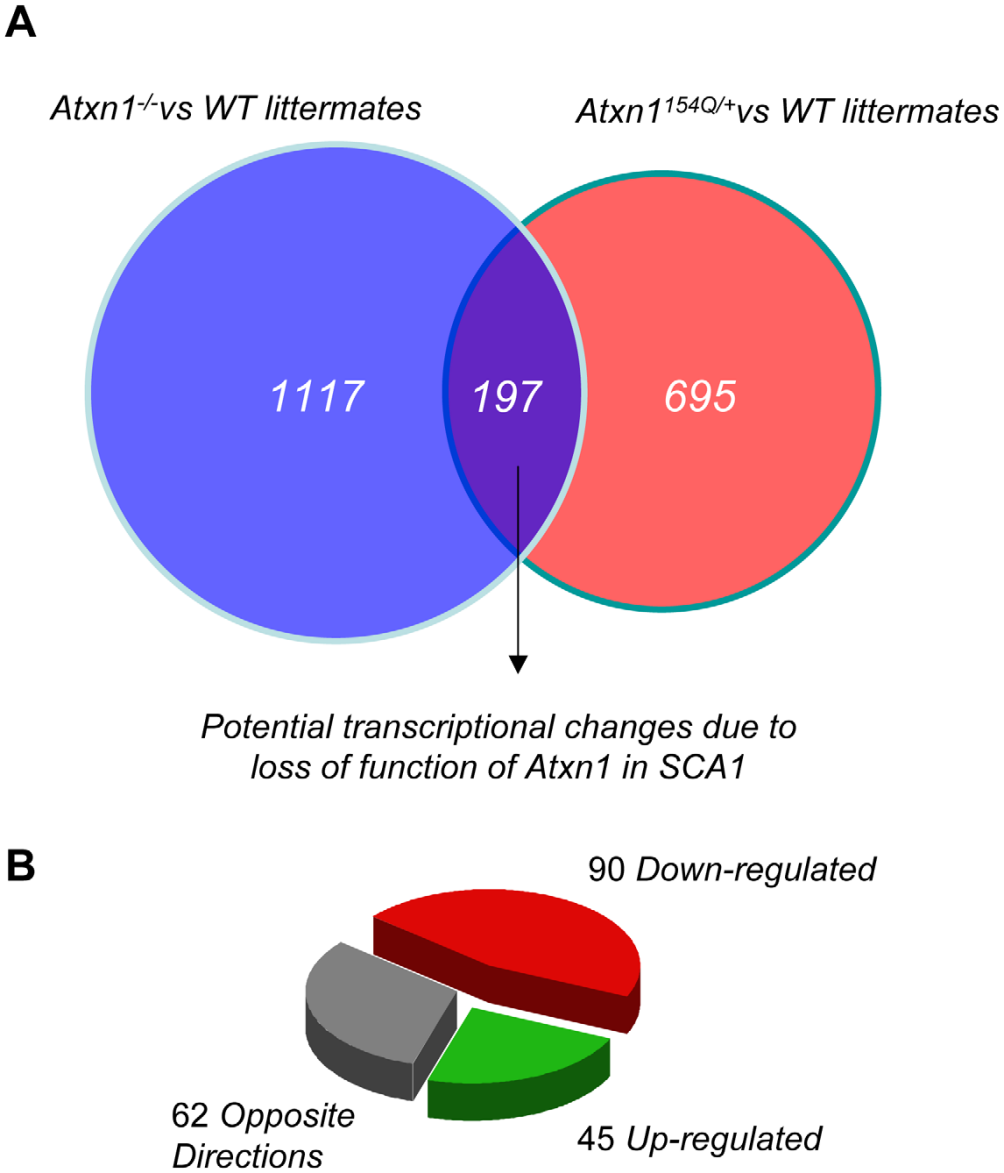
Comparison of transcriptional profiles of *Atxn1^154Q/+^* and *Atxn1^−/−^* cerebella. (A) Venn diagram reporting the number of significant gene expression changes in *Atxn1^−/−^* and *Atxn1^154Q/+^*cerebella. A total of 197 transcripts were significantly altered in both *Atxn1^−/−^* and *Atxn1^154Q/+^*mice, using a p-value of less than 0.01, and a minimal fold change of |±0.1| log_2_. (B) The majority of shared changes (68% of genes or 135 out of 197) went in the same direction.

To identify gene expression changes in SCA1 that might be due to partial loss of function of Atxn1, we surveyed transcriptional changes in *Atxn1* ^−/−^ and *Atxn1^154Q/+^*mouse cerebella using the Affymetrix mouse Exon Array ST 1.0 and searched for shared expression alterations. These arrays potentially enable the detection of even small fold changes due to the existence of multiple probes sets for most transcripts. Early symptomatic (7-week-old) *Atxn1^154Q/+^*mice were used to reflect early changes in SCA1 pathogenesis (Figure 1A). Due to a lack of overt phenotypes in 7-week-old *Atxn1* null mice [13], 16-week-old *Atxn1* ^−/−^ mice were chosen in order to maximize the potential number of gene expression changes detected. Each mutant allele was studied and compared to age-matched wild-type littermates controls.

We observed a highly significant rate of concordance and overlap between cerebellar expression profiles of *Atxn1* ^−/−^ and *Atxn1^154Q^* ^/+^ mice (z  =  11.9485, *P*-value<2.2e-16, Kendall-τ test). Applying a very stringent cutoff value (false discovery rate (FDR)–corrected *P*<0.01, Fold change ≥|±0.1| log_2_), we identified 197 transcripts that are significantly dysregulated in both mouse models (Figure 1A). These 197 transcriptional changes account for 22.1% of all gene expression changes detected in *Atxn1^154Q/+^*cerebella (Figure 1A and Table S1). Remarkably, a majority of the differentially regulated transcripts (135 out of 197, 68.5%) are in the same direction in both mouse models. (Figure 1B). A preponderance of down-regulated genes in both models is observed (90 out of 197), followed by genes up-regulated in both models (45 out of 197), with only less than a third (62 out of 197) altered in an opposite direction (Figure 1B). These data suggest that at least 15.1% (135 out of 892) of the cerebellar transcriptional changes found in the SCA1 knock-in model (*Atxn1^154Q^* ^/+^) could be attributed to loss of *Atxn1* function.

To verify the microarray results, we selected 15 of these shared gene expression changes for independent validation by quantitative real-time reverse transcription polymerase chain reaction (qRT-PCR) using cerebellar RNA samples from an independent set of 16-week-old *Atxn1* ^−/−^ and *Atxn1^154Q/+^*mice, and their respective littermates. Ten out of 15 gene expression changes, or 66.6%, were positively validated in *Atxn1* ^−/−^ mice, while 13 out of 15 genes, or 86.7%, were validated in *Atxn1^154Q/+^*cerebella (Table 1). Most importantly, of the 10 genes validated in *Atxn1* ^−/−^ cerebella, 9 were also validated in *Atxn1^154Q/+^*cerebella (Table 1). Taken together, the comparison of cerebellar microarray studies of *Atxn1* null and polyglutamine-expanded *Atxn1* knock-in mouse models demonstrate that a significant amount of transcriptional changes are shared between these models, supporting the notion that some loss of *Atxn1* endogenous function contributes to disease.

**Table 1:** Real-time qRT–PCR validation of genes commonly altered in *Atxn1^154Q/+^* and *Atxn1^−/−^* cerebella.

Gene symbol	*Atxn1^−/−^ p-value*	*Atxn1^−/−^* Fold Change	*Atxn1^154Q/+^ p-value*	*Atxn1^154Q/+^* Fold Change
*Ifgbp5*	**0.0422**	−1.39	**1.20E-05**	−3.55
*Nab2*	0.7402	−1.04	**0.0004**	−2.03
*Rasal1*	**0.0095**	−1.45	**2.78E-06**	−2.47
*AW551984*	0.1273	2.36	0.8609	−1.12
*Ldb2*	0.5421	1.15	0.2139	−1.36
*Svep1*	**0.0099**	1.27	**0.0003**	−1.49
*Apba2bp*	**0.0288**	1.28	**0.0001**	−1.51
*Synpr*	0.4993	1.26	0.0588	1.71
*Adcyap1r1*	0.1396	1.29	0.7922	1.05
*Robo1*	**0.0002**	1.62	**0.0033**	1.44
*Tgfb3*	**4.76E-05**	1.32	**0.0254**	1.16
*Creb5*	**0.0012**	1.44	**0.0143**	1.38
*Ccnd1*	**4.61E-05**	1.34	**0.0018**	1.23
*Pafah1b3*	**4.09E-07**	1.86	0.1448	1.12
*Tiam2*	**2.09E-05**	1.47	**0.0149**	1.17
*Etv5*	**0.0078**	1.35	0.8350	1.03

P-values for significant genes are represented in bold.

In order to gain insight into the potential molecular pathways commonly affected in *Atxn1* ^−/−^ and *Atxn1^154Q^* ^/+^ mice, we performed Gene Ontology (GO) analysis and pathway analysis based on the Kyoto Encyclopedia of Genes and Genomes (KEGG) (Figures S1, S2, S3, S4, S5, S6, and Table S2). GO analysis revealed some biological functions that are enriched in commonly dysregulated genes. For example, cell junction and synapse, guanyl-nucleotide exchange factor activity and GTPase activity categories were enriched in the common set of up-regulated genes (Figures S1, S2, S3). In the down-regulated gene set, genes encoding for calcium ion binding were the most significantly affected both in *Atxn1^154Q^* ^/+^ and *Atxn1* ^−/−^ cerebella (Figures S4, S5, S6). Using KEGG pathway analysis, among the top enriched categories for genes commonly down-regulated in both *Atxn1* ^−/−^ and *Atxn1^154Q^* ^/+^ mice are the phosphatidylinositol and calcium signaling, Long Term Depression (LTD) associated genes, and Alzheimer's disease pathways (Table S2). These results strongly suggest that loss of Atxn1 results in transcriptional changes that are potentially pathogenic, since in addition to the enrichment for genes involved in neurodegenerative disease, the phosphatidylinositol and calcium signaling pathways are also known to be dysregulated in SCA1 models [26]–[28]. Interestingly, commonly up-regulated genes in *Atxn1* ^−/−^ and *Atxn1^154Q^* ^/+^ mice include genes that are involved in cancer pathways (Table S2). This could potentially point to a novel function of Atxn1 that remains to be clarified in proliferating cells. The categories enriched for genes going in opposite directions between *Atxn1* ^−/−^ and *Atxn1^154Q^* ^/+^ mice involve citrate cycle and ubiquitin-mediated proteolysis. These genes going in opposite directions are of interest, since they might reflect potential differences reflecting the main gain of function mechanism in SCA1 pathogenesis.

### *Atxn1* ^−/−^ cerebella exhibit key pathological gene expression changes present in *staggerer* mice

Given the significant overlap between the transcriptional profiles of *Atxn1^−/−^* and *Atxn1^154Q/+^*cerebella, we were interested in examining whether these transcriptional changes are due to *Atxn1* function being affected in both mouse lines and not simply due to cerebellar dysfunction. Previous studies showed that ATXN1 and RORα interact genetically and biochemically, and deficiency of *Rorα* enhances phenotypes in a transgenic model of SCA1 [20]. Interestingly, we observed a significant overlap between the genetic profiles of *Atxn1* ^−*/*−^ mice, and those common between *staggerer* mice and SCA1 transgenic mice (Table 2) [20], [21]. Most of these shared changes between *staggerer* and SCA1 mice are not present in *Sca7^266Q^* ^/+^ cerebella, a knock-in mouse model for SCA7 [27], suggesting that most of these changes are not due to cerebellar dysfunction or polyglutamine disease in general (Table 2). We were able to confirm some of these changes by real-time quantitative RT-PCR in cerebella from 16-week-old *Atxn1* ^−*/*−^ mice, validating 4 out of 6 transcriptional changes tested (Figure S7). Given the known genetic and physical interaction between ATXN1 and RORα [20], these results could potentially indicate that *Rorα* -dependent transcriptional regulation is altered by loss of *Atxn1* function. In contrast to SCA1 transgenic mice, it is important to note that Rorα levels are not changed in *Atxn1^−/−^* mice [20]. Together, these findings suggest that loss of endogenous function of *Atxn1* results in transcriptional changes that could potentially contribute to cerebellar pathogenesis.

**Table 2:** Rorα-responsive genes altered in SCA1 mouse models and *Atxn1^−/−^* mice.

Gene symbol	*Rora* ^(sg/sg)^ a	*SCA1[82Q]* Tg^b^	*Atxn1* ^−/−^	*Atxn1^154Q^* ^/+^	*Sca7^266Q^* ^/+^ c
*Rora*	down	down			
*Car8*	down	down		down	
*Inpp5a*	down	down	down	down	down
*Slc1a6*	down	down	down		
*Actl6a*	down		down	down	
*Ccna2*	down		down		
*Atp2a2*	down	down	down	down	
*Itpr1*	down	down	down	down	
*Grid2*	down		down		
*Mela*	down		down	down	
*Calb1*	down	down	down	down	down
*Grm1*	down	down	down		
*Nek2*	down		down		
*Id2*	down	down	down	down	down
*Spnb3*	down	down	down		
*Pcp2*	down	down			
*Pcp4*	down	down			down
*Kitl*	down	down		down	
*Sst*	up		up		

^**a**^Gold *et al* 2003 [21].

^**b**^Serra *et al* 2006 [20].

^**c**^Gatchel *et al* 2008 [27].

### Atxn1 and Capicua associate at the promoter regions of genes altered in *Atxn1^−/−^* and *Atxn1^154Q^* ^/+^ cerebella

ATXN1 forms stable complexes *in vivo* with the Capicua homolog (CIC), a transcriptional repressor that exhibits reduced levels in *Atxn1^154Q^* ^/+^ mice [18], [23]. To test the hypothesis that reduced Atxn1-Cic complexes lead to dysregulated gene expression in *Atxn1^154Q/+^*mice, we searched our microarray data for up-regulated genes that are direct targets of Cic. Microarray analysis revealed at least 3 significantly up-regulated genes (False Discovery Rate-corrected *P<0.05*) in *Atxn1^−/−^* cerebella that have been identified as direct targets of Cic-mediated repression, namely *Etv1*, *Etv5* [29], and *Ccnd1* (Fryer and Zoghbi, unpublished data). Interestingly, the microarray data show that *Etv5* and *Ccnd1* are also significantly up-regulated in *Atxn1^154Q/+^*mice cerebella. These results could reflect the fact that Cic protein levels are diminished both in *Atxn1* ^−/−^ and *Atxn1^154Q/+^*mice [18], [23].

**Figure 2:**
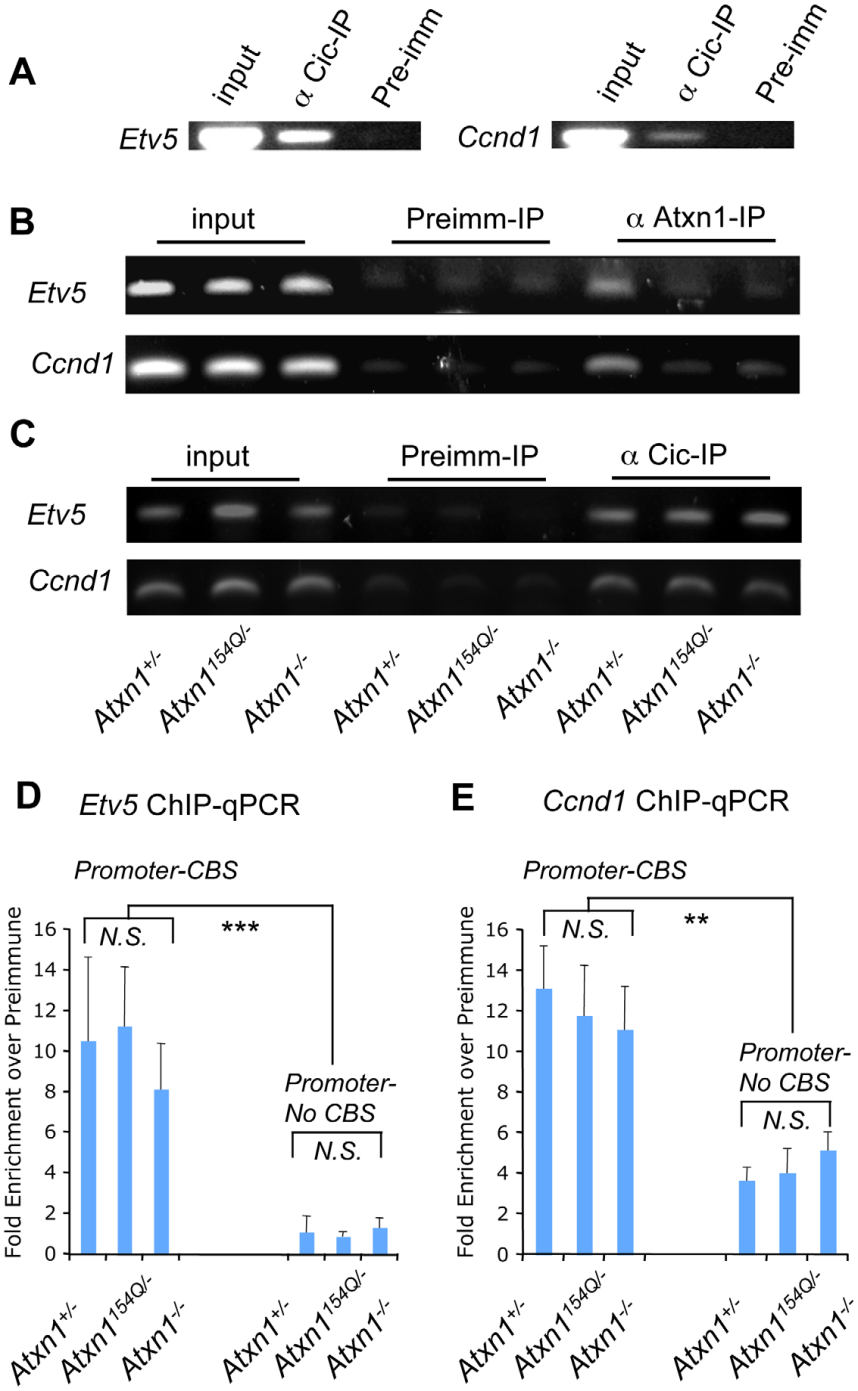
Chromatin immunoprecipitation (ChIP) reveals co-occupancy at the promoters of Cic target genes by Atxn1 and Cic. (A) ChIP using Cic antisera confirmed Cic binding at the promoter of two direct targets of Capicua, *Ccnd1* and *Etv5*, that were up-regulated in the *Atxn1^−/−^* and *Atxn1^154Q/+^* cerebella. (B) ChIP using Atxn1 anti-sera in cerebellar extracts from *Atxn1* ^+/−^, *Atxn1^154Q/−^*, and *Atxn1* ^−/−^ mice reveals a signal in mice expressing only wild-type (*Atxn1* ^+/−^) but not polyQ-Atxn1 (*Atxn1^154Q/−^*) compared to negative controls (pre-immune sera, and *Atxn1* ^−/−^) (C) ChIP as in (B), this time using Cic antibody. In contrast to (B), Cic is present at comparable levels at the target promoters in *Atxn1* ^+/−^, *Atxn1^154Q/−^*, and *Atxn* ^−/−^ mice. All ChIP assays were repeated three times on independent samples, representative results shown. (D) ChIP followed by quantitative PCR (ChIP-qPCR) on the promoter of *Etv5* confirms that the proportion of immunoprecipitated DNA by Cic antibody is comparable in all three genotypes (as seen in C). A region containing two Capicua binding sites (promoter-CBS) of *Etv5* is more enriched by Cic antibody than a region lacking CBSs (promoter-no CBS) compared to preimmune sera. (E) ChIP-qPCR on the promoter of *Ccnd1* also shows similar enrichment of immunoprecipitated DNA by Cic antibody in all three genotypes (as seen in C). ChIP-qPCR using primers designed for a region within 100 bps of the CBS in the *Ccnd1* promoter (which is highly conserved across species) show more Cic binding than primers designed for a poorly conserved region further downstream (∼400 bps) of the CBS at the promoter of *Ccnd1*, compared to preimmune sera. ChIP-qPCR experiments in (D) and (E) were performed in triplicate on three independent sets of samples (3 cerebella per genotype) using SYBR Green Dye. N.S. = not significant, ** p<0.01, *** p<0.005.

Since Atxn1 and Cic form stable complexes *in vivo*, we rationalized that both proteins should bind the promoter regions of target genes if they mediate transcriptional repression together as a complex. To test this possibility, we performed chromatin immunoprecipition analysis, followed by PCR for the promoter regions of *Etv5* and *Ccnd1* (ChIP-PCR). ChIP-PCR analysis using antibodies against Cic confirmed that Cic is present on the promoters of *Etv5* and *Ccnd1 in vivo* (Figure 2A). To determine if Atxn1 binds to the promoters of Cic targets and if the binding is altered due to polyglutamine expansion, we prepared cross-linked chromatin from mice expressing one wild-type Atxn1 allele (*Atxn1* ^+/−^) and compared it to mice expressing one polyglutamine-expanded Atxn1 allele (*Atxn1^154Q^* ^/−^). We used *Atxn1* ^−/−^ mice as a negative control for testing Atxn1 antibody specificity. As predicted, in chromatin extracts prepared from *Atxn1^+/^* ^−^ cerebella and immunoprecipitated using Atxn1 antibody, wild-type Atxn1 was detected on the promoters of *Etv5* and *Ccnd1* (Figure 2B). In contrast, we could not detect any specific signal for Atxn1[154Q] above background levels in *Atxn1^154Q/−^* cerebellar chromatin immunoprecipitations using Atxn1 antibodies (compared to *Atxn1* ^−/−^ and pre-immune sera controls) (Figure 2B). These data suggest that there is minimal association of polyglutamine-expanded Atxn1[154Q] to the promoters of target genes *in vivo*. Alternatively, it is possible that a conformational change in Atxn1[154Q] renders it inaccessible for immunoprecipitation, resulting in reduced signal.

Given that Atxn1[154Q]-specific signal is not detected on the promoters of *Etv5* and *Ccnd1*, we next asked if Cic protein can be detected at these promoters in *Atxn1^154Q/−^* mice using Cic antisera. We detected Cic binding to the promoter regions of *Etv5* and *Ccnd1* (Figure 2C) on all genotypes tested (*Atxn1^+/^* ^−^, *Atxn1^154Q/−^* and *Atxn1^−/−^*), despite the fact that Cic protein levels are reduced in *Atxn1^−/−^* and *Atxn1^154Q/−^* mice [18], [23]. To better quantify potential differences in Cic binding between the different genotypes, we performed ChIP followed by quantitative PCR. Primer sets designed for conserved regions in the promoters of *Etv5* and *Ccnd1* that contain or are adjacent to Cic binding sites (CBS) (TGAATGAA or TGAATGGA) were able to amplify with no significant difference in all three genotypes (*Atxn1^+/−^*, *Atxn1^154Q/−^* and *Atxn1^−/−^*), both for *Etv5* and *Ccnd1* (Figure 2D–2E). To test for the specificity of Cic-binding to its consensus sequences, primers were designed for regions lacking predicted Cic-binding sites sequences, either upstream or downstream of the CBS-containing regions in *Etv5* and *Ccnd1* promoters (Figure 2D–2E). As expected, quantitative PCR for these regions show less binding relative to the corresponding positive regions of *Etv5* and *Ccnd1* by quantitative PCR. These findings suggest that although Cic still binds the promoter, its function in repression is less efficient in the absence of wild-type Atxn1 or that Atxn1L partially compensates for the loss of Atxn1 at the promoters. The gene expression and ChIP-PCR data suggest that polyglutamine-expanded Atxn1 is less efficient in Cic-dependent repression. More importantly, they provide evidence for a model in which loss of Atxn1/Cic function can result in transcriptional dysregulation, contributing to SCA1 pathogenesis.

### *Atxn1L^dp/^* ^+^ rescues Cic levels in *Atxn1^−/−^* mice by forming functional Atxn1L-Cic complexes

**Figure 3:**
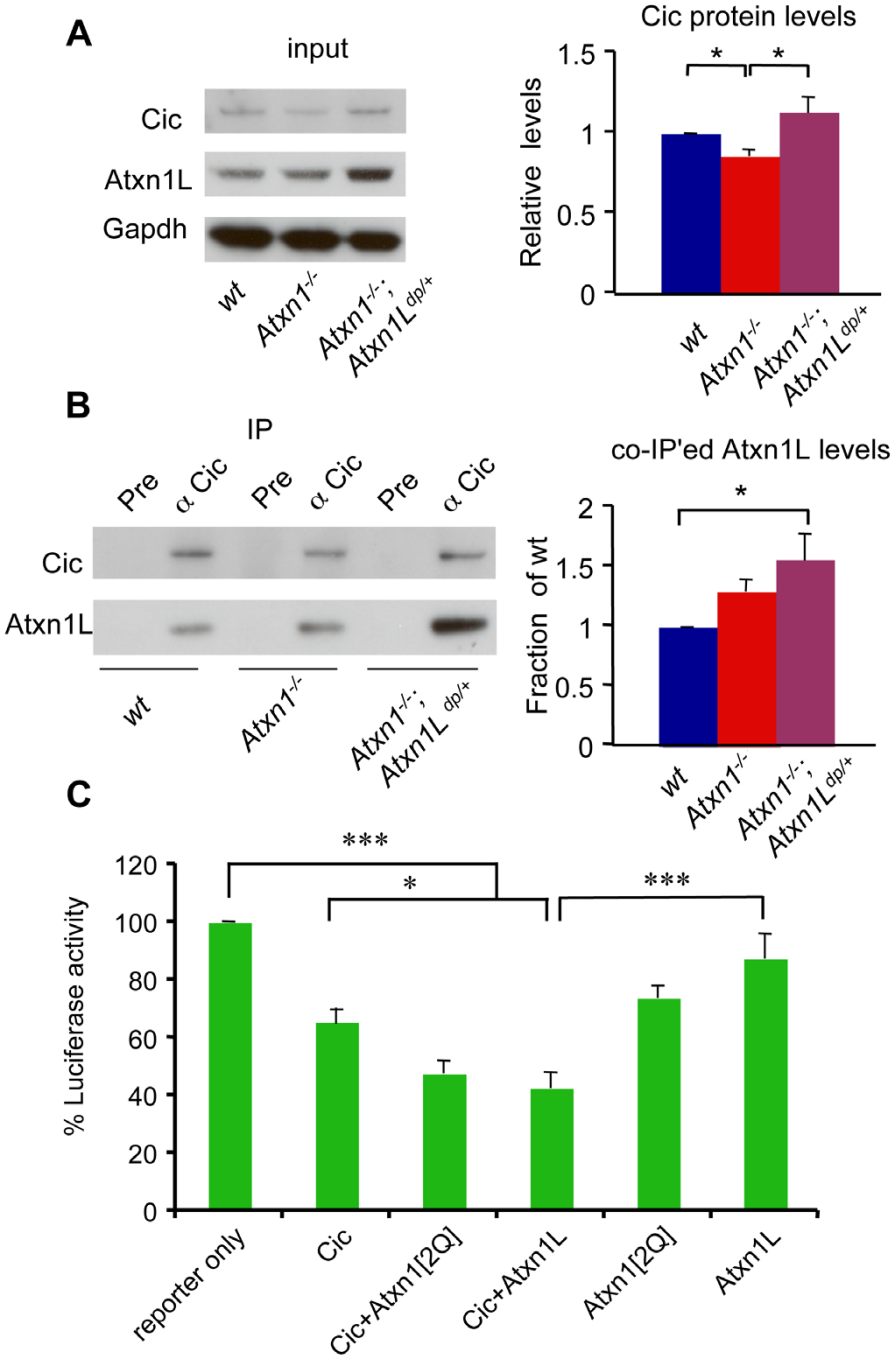
*Atxn1L^dp^* stabilizes Cic protein levels in *Atxn1^−/−^* mice by enhancing Atxn1L-Cic complex formation. (A) Western blot analysis shows that overexpression of *Atxn1L* rescues the reduced Cic levels in *Atxn1^−/−^* mice (* p<0.05). (B) Co-immunoprecipitation of Atxn1L using Cic antibodies show that, despite the reduced levels of Cic protein in *Atxn1^−/−^* cerebella, the relative fraction of Atxn1L co-immunoprecipitated with Cic in *Atxn1^−/−^* protein extracts was greater than in wild-type cerebella. Atxn1L overexpression further increased the levels of Atxn1L-Cic co-immunoprecipitation in *Atxn1^−/−^*; *Atxn1L^dp/+^* mice. Images show representative blots and the quantification of three independent experiments. Error bars in graphs represent +/−SEM. * p<0.05. (C) To determine if Atxn1L-Cic complexes are functional, we measured the transcriptional effect of Cic, Atxn1 and Atxn1L on the expression of a luciferase reporter construct containing a tandem array of Cic binding sites (CBS). Luciferase activity is expressed as fraction of the activity when the reporter is expressed alone (100%). Co-transfection of Cic and Atxn1[2Q]) results in synergistic co-repression of this luciferase reporter (Cic+Atxn1[2Q]). Interestingly, co-transfection of constructs expressing Cic and Atxn1L (Cic+Atxn1[2Q]) results in synergistic repression of the reporter similar to co-transfection of Cic and wild-type Atxn1. These results suggest that Atxn1L-Cic complexes are functional in Capicua-dependent repression. Assays were performed in duplicate in 5 independent experiments. Error bars in graph represent +/− SEM, *p<0.05,***p<0.005.

Given that mild overexpression of the Atxn1 paralog, Atxn1L, can displace both wild-type and polyglutamine-expanded Atxn1 from large native complexes in a dose-dependent manner, we wondered if Atxn1L could partially replace Atxn1 as a Cic binding partner, especially because Atxn1L interacts with Cic in wild-type cerebellum [19], [24]. This led us to propose that in addition to competing with Atxn1[154Q] in the large native complexes [24], increased Atxn1L levels can suppress a putative loss of *Atxn1* function in *Atxn1^154Q^* ^/+^ mice by directly substituting for Atxn1 in Cic-containing complexes. To test this possibility, we generated *Atxn1^−/−^* and *Atxn1^−/−^*; *Atxn1L^dp/+^* littermates, and performed western blot analysis for Cic. As shown in Figure 3A, Atxn1L overexpression results in restoration of Cic levels back to wild-type levels in *Atxn1^−/−^* cerebellum. We then tested if overexpression of Atxn1L increased formation of Atxn1L-Cic complexes, thus stabilizing Cic. For this, we performed co-immunoprecipitation studies using Cic antibody on cerebellar extracts of *Atxn1^−/−^* mice with or without the *Atxn1L^dp^* allele, followed by western blot analysis for Atxn1L. As expected, Atxn1L co-immunoprecipitates with Cic in wild-type cerebella (Figure 3B). However, despite the reduced levels of Cic protein in *Atxn1^−/−^* cerebella (Figure 3A and [18]), the relative fraction of Atxn1L co-immunoprecipitated with Cic in *Atxn1^−/−^* protein extracts was greater than in wild-type cerebella (Figure 3B). Atxn1L overexpression further increased the levels of Atxn1L-Cic co-immunoprecipitation in *Atxn1^−/−^*; *Atxn1L^dp/+^* mice (Figure 3B). These results suggest that Atxn1L overexpression enhances the formation of Atxn1L-Cic complexes in *Atxn1^−/−^* mice, thus stabilizing Cic protein levels.

We further asked whether Atxn1L-Cic complexes were proficient in Cic-dependent transcriptional repression. For this, we co-transfected HEK293T cells with a luciferase reporter construct containing a tandem array of Cic binding sites [18], [29], along with Atxn1L and Cic-expressing plasmids. Consistent with previous studies [18], co-transfection of Cic and Atxn1 resulted in synergistic repression of the Cic-responsive luciferase reporter (Figure 3C). Interestingly, co-transfection of constructs expressing Cic and Atxn1L resulted in synergistic repression of the reporter similar to co-transfection of Cic and wild-type Atxn1 (Figure 3C). These results strongly suggest that Atxn1L-Cic and Atxn1-Cic complexes are functionally redundant in Cic-dependent transcriptional repression. Thus, we conclude that mild overexpression of *Atxn1L* in *Atxn1^−/−^* mice might partially rescue a loss of *Atxn1* endogenous function related to reduced Atxn1-Cic complexes.

### *Atxn1L^dp/^* ^+^ restores some gene expression alterations in *Atxn1* ^−/−^ mice

**Figure 4:**
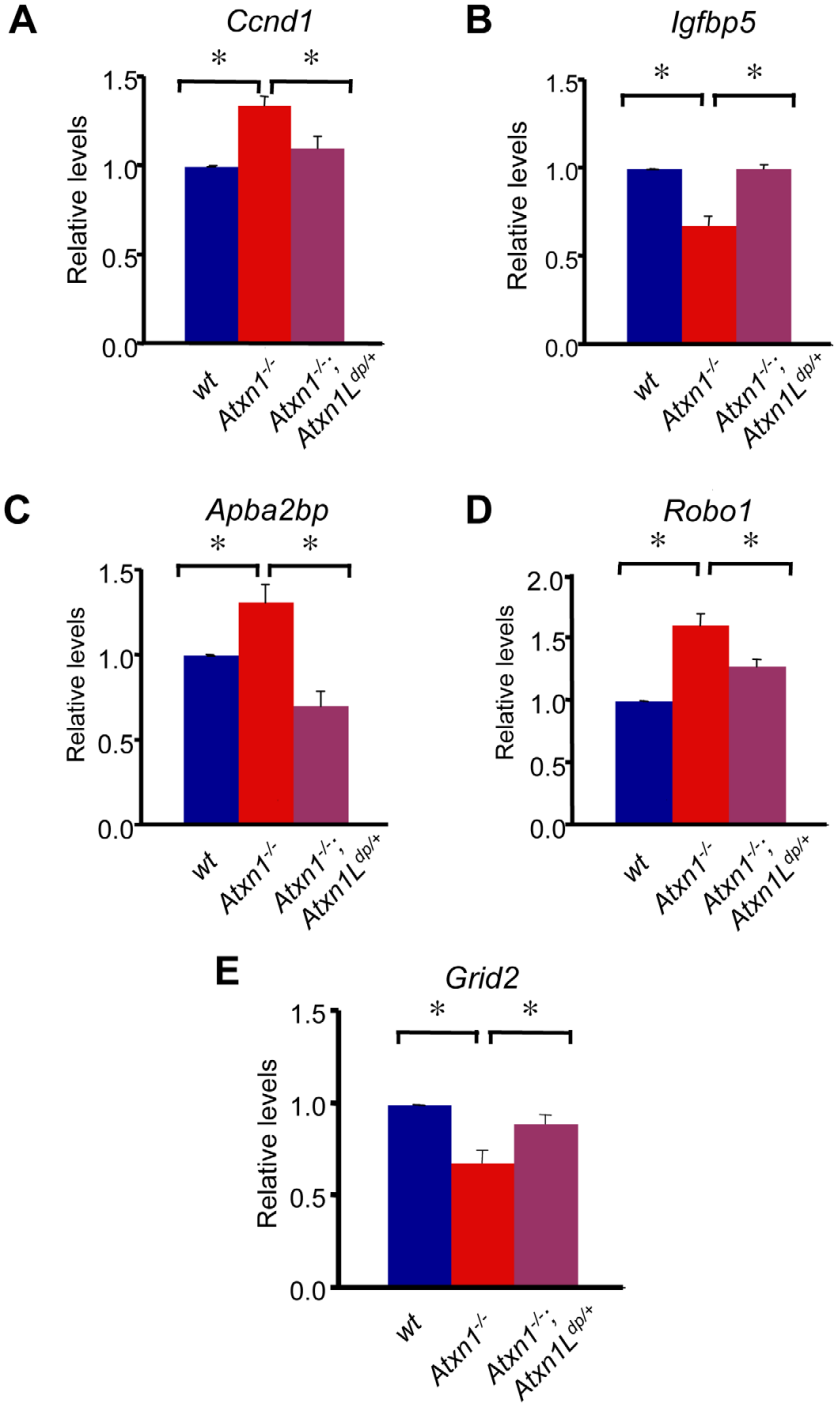
Mild overexpression of *Atxn1L* restores expression levels of some genes altered in *Atxn1^−/−^* cerebella. Real-time quantitative RT-PCR on RNA extracted from wild-type (n = 8), *Atxn1* ^−/−^ (n = 10) and *Atxn1^−/−^*; *Atxn1L^dp/+^* cerebella (n = 6) for (A) *Ccnd1*, (B) *Igfbp5*, (C) *Apba2bp*, (D) *Robo1*, and (E) *Grid2* reveals that these transcripts were restored close to wild-type levels in *Atxn1^−/−^*; *Atxn1L^dp/+^* cerebellum. Real-time qRT-PCR experiments were performed in triplicate for each sample. Error bars represent +/−SEM, *p<0.05.

Given that mild *Atxn1L* overexpression rescues Cic levels in *Atxn1^−/−^*; *Atxn1L^dp/+^* mice, we predicted that *Atxn1L* should rescue some of the transcriptional changes in *Atxn1^−/−^* cerebella if it can functionally substitute for *Atxn1*. To test this, we performed qRT-PCR on cerebellar RNA isolated from *Atxn1^−/−^*; *Atxn1L^dp/+^* mice and *Atxn1^−/−^* littermates at 16 weeks of age. We focused our qRT-PCR analysis on the 9 genes that are commonly dysregulated in *Atxn1^154Q/+^* and *Atxn1^−/−^* cerebella (Table 1), and also on the 4 down-regulated Rorα targets validated by qRT-PCR in *Atxn1^−/−^* cerebellum (Figure S7). Interestingly, 5 out of 13 genes tested (*Ccnd1*, *Igfbp5*, *Apba2bp*, *Robo1* and *Grid2*) were partially or completely restored back to wild-type levels in *Atxn1^−/−^*; *Atxn1L^dp/+^*, compared to *Atxn1^−/−^* cerebella (Figure 4A–4E). *Ccnd1*, one of the up-regulated genes that is potentially a direct target of Atxn1-Cic complexes (Figure 4A), and *Igfbp5*, an early key pathogenic marker in SCA1 disease (Figure 4B), were among the genes rescued by *Atxn1L* overexpression in *Atxn1^−/−^* mice. Additionally, the Rorα target *Grid2* is also significantly rescued in *Atxn1^−/−^*; *Atxn1L^dp^* ^/+^ mice. Thus, mild overexpression of *Atxn1L in vivo* results in partial rescue of several transcriptional changes related to SCA1 pathogenesis in a loss-of-function model of *Atxn1*. Since Atxn1L binds to transcriptional regulators that interact with ATXN1, such as CIC and SMRT-NCoR, it is possible that Atxn1L rescues transcriptional changes by functionally replacing polyglutamine-expanded Atxn1 in these transcriptional complexes.

### Partial rescue of behavioral defects in *Atxn1* ^−/−^ mice by mild overexpression of *Atxn1L*

Although *Atxn1^−/−^* mice do not exhibit overt ataxia phenotypes or progressive neurodegeneration, they do exhibit a variety of neurological deficits ([13] and Figures S8, S9, S10, S11). Interestingly, *Atxn1^−/−^* mice exhibit deficits in spatial learning and memory, and in motor learning and coordination, phenotypes shared with *Atxn1^154Q/+^*mice ([13] and Figures S10 and S11). This raises the possibility that partial loss of Atxn1 function of could also contribute to these phenotypes in *Atxn1^154Q/+^*mice. The fact that the *Atxn1L^dp^* allele can suppress behavioral phenotypes in *Atxn1^154Q/+^*mice [24] also raises the possibility that Atxn1L suppresses SCA1 pathogenesis by functionally replacing those Atxn1 functions altered by polyglutamine-expanded Atxn1.

**Figure 5:**
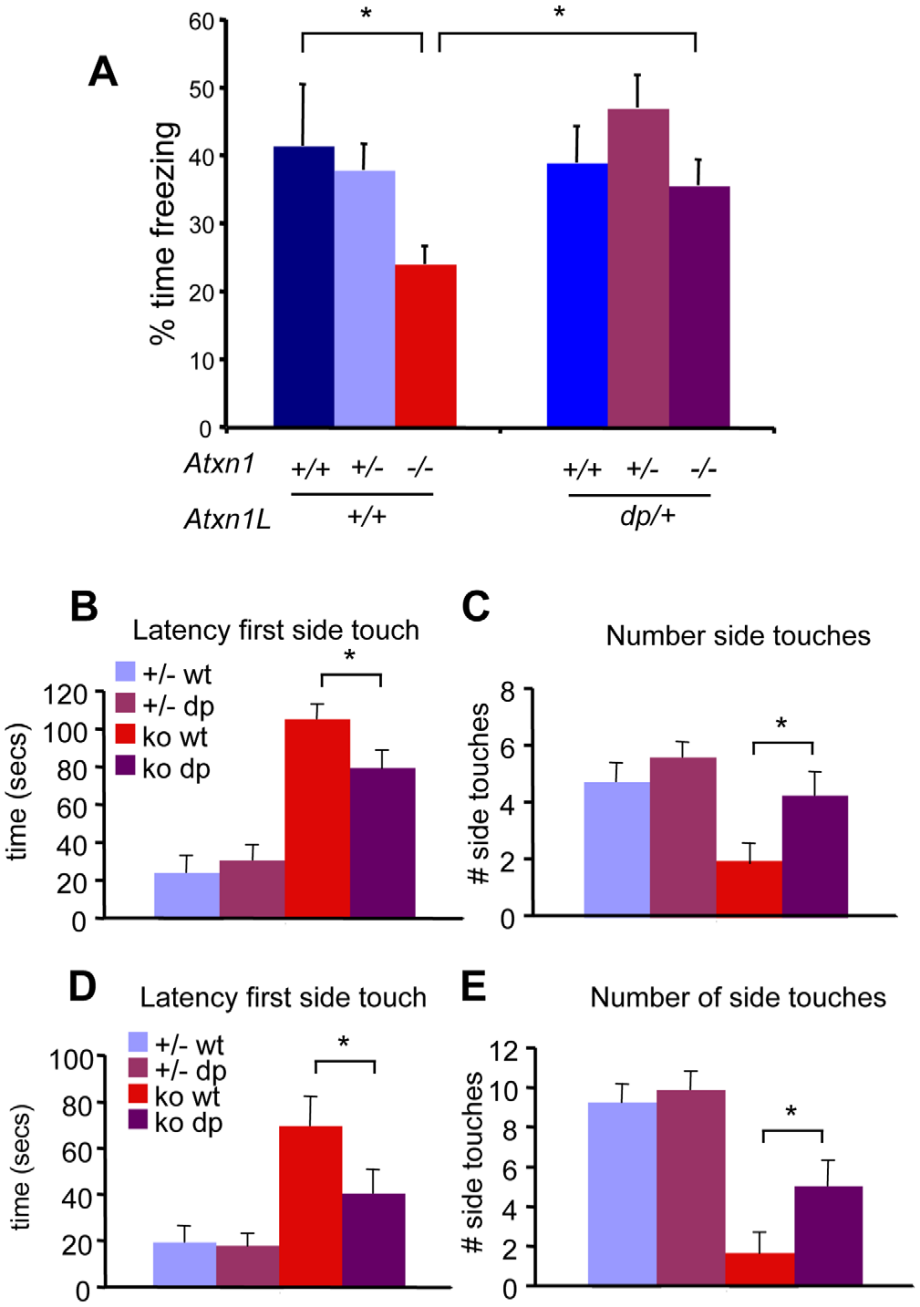
Atxn1L^dp^ suppresses the behavioral deficits in *Atxn1^−/−^* mice. We assessed the effects of mild overexpression of Atxn1L on *Atxn1^−/−^* phenotypes (A) Mice of the following genotypes: *Atxn1^+/+^* (n = 5), *Atxn1* ^+/−^ (n = 12), *Atxn1* ^−/−^ (n = 9), *Atxn1* ^+/+^; *Atxn1L^dp/^* ^+^(n = 10), *Atxn1* ^+/−^; *Atxn1L^dp/^* ^+^(n = 17), and *Atxn1* ^−/−^; *Atxn1L^dp/^* ^+^(n = 12), were tested at 8 weeks for contextual conditioned fear. *Atxn1^−/−^* mice exhibited significantly reduced freezing behavior in the contextual fear-conditioning test compared to wild-type and *Atxn1^+/−^* littermates. However, *Atxn1^−/−^* mice carrying the *Atxn1L* duplication performed significantly better than *Atxn1^−/−^* littermates in this task (p<0.05). Wild-type and *Atxn1^+/−^* mice expressing the *Atxn1L^dp^* allele performed similarly to wild-type and *Atxn1^+/−^* mice without the *Atxn1L* duplication. (B–E) An independent cohort of mice was generated to test the effects of *Atxn1L duplication* on the motor deficits of *Atxn1^−/−^* mice. *Atxn1* ^+/−^ (n = 16), *Atxn1* ^+/−^; *Atxn1L^dp/^* ^+^(n = 23), *Atxn1* ^−/−^ (n = 14), and *Atxn1* ^−/−^; *Atxn1L^dp/^* ^+^ mice (n = 17) were tested on the dowel and wire hang paradigms at 8 weeks. The latency of *Atxn1^−/−^* mice to reach the side for the first time was increased compared to *Atxn1^+/−^* and *Atxn1^+/−^*; *Atxn1^dp/+^* littermates on the rod (B); they also walked off the rod fewer times in the 120 s interval (C). In contrast, *Atxn1^−/−^*; *Atxn1L^dp/+^* mice took less time to walk off the dowel (B), and they also crossed the dowel more times in 120 s than *Atxn1^−/−^* littermates (C). In the wire hang test, *Atxn1^−/−^* mice showed increased latency to reach the sides of the wire compared *Atxn1^+/−^* and *Atxn1^+/−^*; *Atxn1L^dp/+^*controls (D). Additionally, *Atxn1^−/−^* mice reached the sides fewer times than control littermates (E). In contrast, *Atxn1^−/−^* mice overexpressing *Atxn1L* exhibited marked reduction in the time for the first touch (D), and increased number of side touches in the 120 s interval, when compared to *Atxn1^−/−^* mice (E). Error bars in graphs represent +/− SEM, *p<0.05.

To investigate whether *Atxn1L* and *Atxn1* are functionally redundant *in vivo*, we tested if the *Atxn1L^dp^* allele can ameliorate behavioral defects in *Atxn1^−/−^* mice. We focused on two characteristic phenotypes clinically associated with SCA1 disease: cognitive deficits (learning and memory) and deficits in motor coordination and balance [30], [31]. We performed the conditioned fear paradigm on *Atxn1^−/−^* mice carrying the *Atxn1L^dp^ allele* and compared them to *Atxn1^−/−^* littermates. As shown in Figure 5A, and in agreement with previous data [32], *Atxn1^−/−^* mice exhibited significant deficits, as determined by reduced freezing behavior, in the contextual fear-conditioning test compared to wild-type and *Atxn1^+/−^* littermates. Wild-type and *Atxn1^+/−^* mice expressing the *Atxn1L ^dp^* allele performed similarly to the controls (Figure 5A). Interestingly, the duplication of *Atxn1L* rescued the freezing behavior due to loss of *Atxn1* (compare *Atxn1* ^−/−^ mice to *Atxn1^−/−^*; *Atxn1L^dp/+^* mice; Figure 5A).

Having shown that mild overexpression of *Atxn1L* rescues Pavlovian contextual learning in *Atxn1^−/−^* mice, we then wanted to assess the effects of increased Atxn1L levels on motor coordination and balance deficits in *Atxn1^−/−^* mice, another phenotype common to SCA1 mouse models. In order to discriminate between motor learning impairments and motor coordination deficits, we chose to use the dowel rod and wire hang tests. These paradigms do not rely on consecutive daily training, as does the rotating rod test, therefore we selected them in order to discern motor coordination and balance defects from cerebellar learning deficits [33], [34]. We generated an independent cohort of *Atxn1^−/−^* mice and *Atxn1^−/−^*; *Atxn1L^dp/+^* mice, with *Atxn1^+/−^* and *Atxn1^+/−^*; *Atxn1^dp/+^* littermates as controls, and tested them at 8 weeks of age. We measured the latency to reach the side (first touch) and frequency of walking off the rod (number of side touches in 120 seconds) (Figure 5B and 5C). All genotypes tested remained on the dowel for the maximum time (data not shown), but *Atxn1^−/−^* mice moved much less than *Atxn1^+/−^* and *Atxn1^+/−^*; *Atxn1^dp/+^* littermates on the rod. The latency of *Atxn1^−/−^* mice to reach the side for the first time was increased (Figure 5B), and consequently they walked off the rod fewer times (Figure 5C). It is noteworthy that *Atxn1* ^−/−^ mice are active and travel the same distance as wild-type littermates the open field analysis (Figure S5). Thus the hesitancy to move on the dowel suggests that in addition to learning and memory deficits, *Atxn1^−/−^* mice have balance or motor coordination impairments, evident at an early age (8-week-old). In contrast, *Atxn1^−/−^* mice carrying the *Atxn1L^dp^* allele take less time to walk off the dowel (Figure 5B), and they also crossed the dowel more times in 120 s than *Atxn1^−/−^* littermates (Figure 5C). Thus, mild *Atxn1L* overexpression partially rescues the dowel phenotype caused by loss of *Atxn1*.

The wire-hang paradigm assesses motor coordination and grip strength [34]. In this paradigm, mice are hanging onto the center of an elevated wire from their forepaws, and they need to get to the sides for relief, which requires coordination and normal strength. We measured the time and frequency to reach the sides in a 120 s interval, and found no significant falling off the wire in *Atxn1^−/−^* mice compared to the control littermates, suggesting that they have reasonable grip strength (data not shown). However, *Atxn1^−/−^* mice showed increased latency to reach the sides for the first time compared *Atxn1^+/−^* and *Atxn1^+/−^*; *Atxn1L^dp/+^* controls (Figure 5D). Additionally, *Atxn1^−/−^* mice reached the sides fewer times than control littermates (Figure 5E). In contrast, *Atxn1^−/−^* mice overexpressing *Atxn1L* exhibited marked reduction in the time for the first touch and increased number of side touches in the 120 s interval, when compared to *Atxn1^−/−^* mice (Figure 5D and 5E). Taken together, these behavioral data demonstrate that a 50% increase in the levels of AtxnlL is sufficient to partially rescue several behavioral deficits caused by loss of Atxn1 function. Furthermore, this rescue correlates with the molecular data, demonstrating that *Atxn1L* is a functional homolog of *Atxn1 in vivo*.

## Discussion

Recently, we proposed the possibility that in addition to toxic gain-of-function due to polyglutamine-expanded ATXN1, a concomitant partial loss of ATXN1 function might contribute to SCA1 pathogenesis [23]. It is challenging to establish the extent of the contribution of a potential loss-of-function mechanism to SCA1 pathogenesis in models carrying the mutant protein, since the severe gain-of-function effects might mask any subtle loss-of-function component, thus confounding the interpretation of the results. In the present study, we sought to distinguish between gain- and loss-of-function mechanisms by focusing on transcriptional defects in *Atxn1^−/−^* mice, and comparing them to the knock-in model of SCA1, *Atxn1^154Q/+^* mice. Using this approach, we identified several molecular changes that could be attributable to loss of ATXN1 function in SCA1.

We found that loss-of-function of *Atxn1* in mice is sufficient to cause many transcriptional changes common to the *Atxn1^154Q/+^* knock-in mice, a model of SCA1 that faithfully replicates many features of the disease, and with *SCA1[82Q]* transgenic mice. It has been reported that ATXN1 interacts with several factors involved in transcriptional regulation, including CIC, SMRTER, HDAC3, Gfi1 and RORα [15], [18]–[20]. Therefore, these shared expression changes might be indicative of altered transcriptional functions of ATXN1 in SCA1 pathogenesis. Furthermore, we showed that a majority of the shared transcriptional changes go in the same direction in both *Atxn1^−/−^* and *Atxn1^154Q/+^* mice, strongly arguing that part of the transcriptional dysregulation in SCA1 might be explained by a partial loss-of-function of Atxn1. The up-regulation of direct targets of Atxn1-Cic provides evidence for this concept. Another important finding of this study is that there are many transcriptional changes that are unique to the *Atxn1^154Q/+^* model, which could potentially be related to toxicity of polyglutamine-expanded Atxn1.

We propose that a combination of toxic gain-of-function and mild loss-of-function mechanisms contribute to SCA1 pathogenesis, with the partial loss-of-function of ATXN1 being sufficient to cause some transcriptional changes that are pathogenic in the cerebellum. Previous studies using microarray analysis reported on the down-regulation of the dopamine receptor D2 (*Drd2*) in *Atxn1^−/−^* mouse cerebella [35]. However, with the exception of a couple of genes (e.g. *Pafahb3*, *Sp1*), we were unable to find extensive overlap between the changes reported by Goold *et al.* and the microarray analysis presented in this study. The differences in genetic background, microarray platform, and the age of the *Atxn1* ^−/−^ animals (5-week-old) in the Goold *et al.* studies [35], compared to 16 weeks in our studies, are likely to contribute to the minimal overlap in gene expression changes in the two studies.

Bioinformatics analyses of the genes commonly altered in *Atxn1^−/−^* and *Atxn1^154Q/+^* cerebella show enrichment for categories associated with pathological pathways involved in neurodegeneration (Alzheimer's disease), and also pathways previously implicated in pathogenesis both in knock-in and transgenic SCA1 mouse models, such as the phosphatidylinositol and calcium signaling pathways [26]–[28]. These results strongly suggest that *Atxn1* ^−/−^ mice have dysfunctional cerebella due to a loss of endogenous Atxn1 function. We also found that *Atxn1^−/−^* mice share significant overlap in cerebellar transcriptional profiles with *staggerer* mice, which have a spontaneous loss-of-function mutation in the gene encoding the transcription factor Rorα. Rorα-regulated genes involved in calcium signaling (*Itpr1* and *Calb1*) and glutamatergic signaling (*Grm1* and *Slc1a6*) are significantly down-regulated in *Atxn1* ^−/−^ cerebellum, as determined by microarray analysis and real-time qRT-PCR. It is noteworthy that loss-of-function mutations in several of these genes result in ataxic phenotypes (e.g. *Itpr1*, *Slc1a6*, and *Grm1*) [36]–[38], raising the possibility that simultaneous down-regulation of several of these genes could contribute to the motor coordination impairments observed in *Atxn1* ^−/−^ mice. *Rorα* mRNA transcript and protein levels appear normal in *Atxn1* ^−/−^ cerebellum [20], ruling out that changes in Rorα targets are due to reduced Rorα protein levels in *Atxn1* ^−/−^ mice. Since ATXN1 and Rorα physically interact via Tip60, it is conceivable that loss of Atxn1 affects Rorα-dependent transcription directly [20]. Altogether, these data support two important conclusions: first, that *Atxn1* ^−/−^ cerebellum exhibits pathological molecular changes, even in the absence of progressive neurodegeneration, and second, that transcriptional changes in the loss-of-function model of *Atxn1* could identify endogenous pathways that might also be altered by the expression of mutant Atxn1.

We previously described a reduction of Atxn1-Cic complexes in *Atxn1^154Q/+^*cerebella, with Atxn1[154Q] favoring the formation of enhanced toxic gain-of-function complexes with RBM17 [23]. It is interesting that among the genes up-regulated both in *Atxn1^−/−^* and *Atxn1^154Q/+^*cerebella, we identified two potential direct targets of Cic-dependent repression, *Ccnd1* and *Etv5*, the genes encoding for Cyclin D1 and Ets variant 5, respectively ([29] and J. Fryer, unpublished data). We demonstrated that wild-type Atxn1 and Cic are bound to the promoter regions of *Ccnd1* and *Etv5*. Interestingly, we failed to detect mutant Atxn1[154Q] on these promoters in mice only expressing expanded Atxn1 (*Atxn1^154Q/−^*). One interpretation of this result is that Atxn1[154Q] has diminished association to the promoters, resulting in reduced Atxn1-Cic dependent repression. Alternatively, it is possible that polyglutamine-induced conformational changes make the Atxn1[154Q]-Cic complexes less accessible for antibody recognition, resulting in reduced chromatin immunoprecipitation. Irrespective of the basis of the inability to detect Atxn1[154Q] binding on these promoters, the data strongly suggest that polyglutamine-expanded Atxn1 and Cic have reduced transcriptional repression function on these specific promoters *in vivo*. These results provide a mechanistic explanation on how diminished Atxn1-Cic function can contribute to transcriptional defects in SCA1.

**Figure 6:**
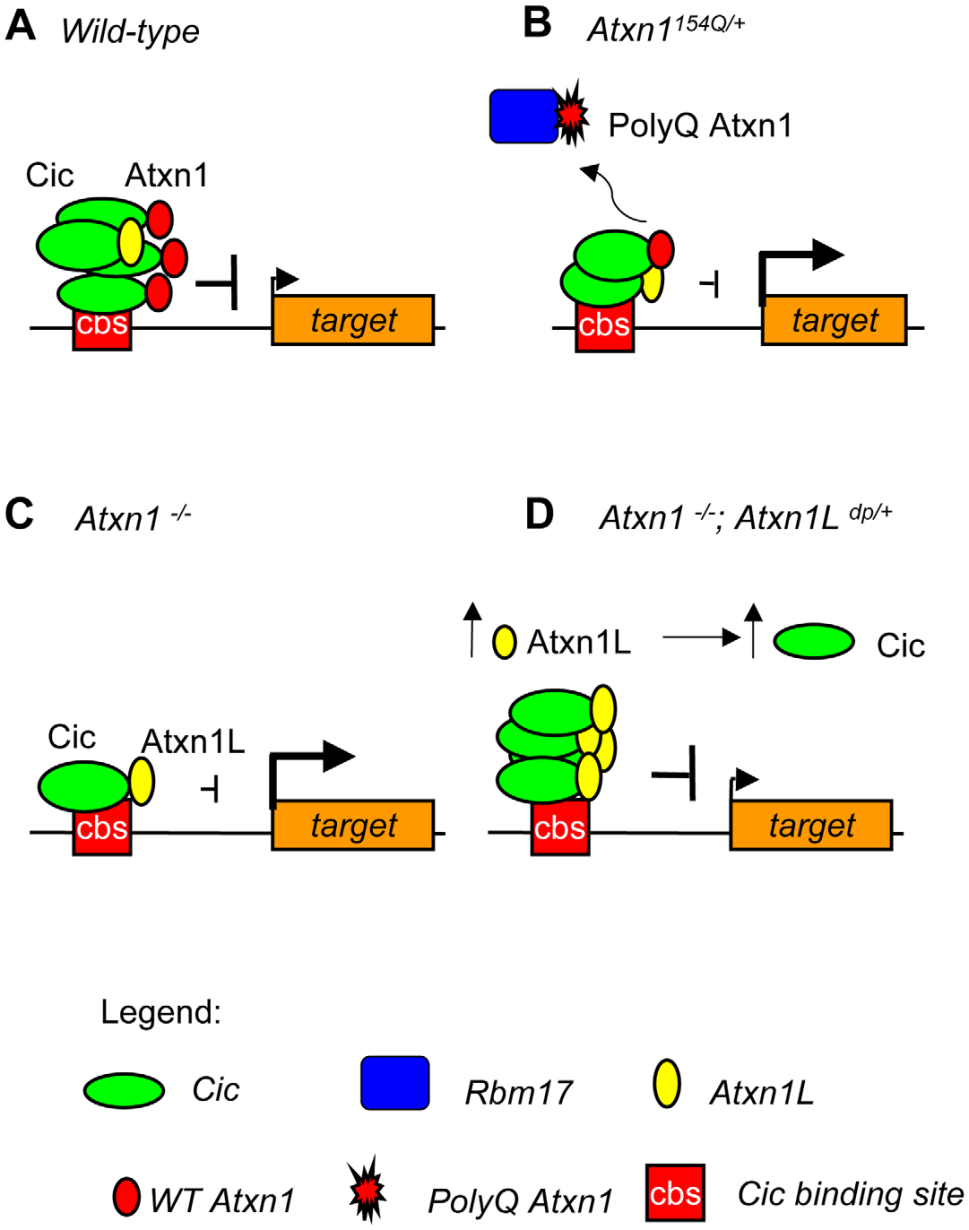
Model of Atxn1L rescue through Cic stabilization in *Atxn1^−/−^* mice. (A) In wild-type mice, Atxn1-Cic and Atxn1L-Cic complexes bind the promoters of target genes and repress them effectively. (B) In mice expressing polyglutamine-expanded Atxn1, mutant Atxn1 associates preferentially with Rbm17, while the decrease in Atxn1-Cic complexes destabilizes Cic and reduces its levels at the promoters, thus leading to de-repression of its target genes. (C) A similar Cic destabilization occurs in the absence of wild-type Atxn1 in *Atxn1^−/−^* mice, also resulting in increased expression of target genes. (D) When *Atxn1L* is moderately overexpressed in *Atxn1^−/−^* mice, it stabilizes Cic levels by forming functional Atxn1L-Cic complexes that can substitute for Atxn1-Cic at the promoters, thus rescuing target gene repression. We propose that this mechanism might also act to rescue loss-of-function of *Atxn1* in *Atxn1^154Q/+^*; *Atxn1L^dp/+^* mice.

Mild overexpression of the evolutionarily conserved gene *Atxn1L* partially suppresses the neuropathology caused by polyglutamine-expanded ATXN1 in flies and mice [19], [24]. Increased Atxn1L levels induce the sequestration of polyglutamine-expanded Atxn1 into nuclear inclusions, leading to a proposed model in which Atxn1L suppresses toxicity by displacement of mutant Atxn1 from its major endogenous complexes that contain Cic [24]. In the present study, we show an additional mechanism contributing to this rescue, by demonstrating that mild overexpression of *Atxn1L* suppresses several molecular and behavioral phenotypes in *Atxn1^−/−^* mice, potentially by replacing Atxn1 in Cic-containing complexes (Figure 6). The motor coordination and learning deficits suppressed by *Atxn1L* are common to both *Atxn1^−/−^* and polyglutamine-expanded *Atxn1^154Q^* ^/+^ mouse models. Therefore, these data provide evidence for an additional mechanism in which *Atxn1L* can functionally compensate for a partial loss of *Atxn1* function to suppress SCA1 pathogenesis. Although our studies demonstrate that *Atxn1L* is a functional homolog of *Atxn1* in Cic-mediated transcriptional repression, we cannot rule out that *Atxn1L* overexpression can restore other yet to be determined *Atxn1*-related functions not addressed in these studies. In sum, based on our previous data and this study, we propose that partial loss of ATXN1 function actively contributes to SCA1 pathogenesis as part of a two-pronged mechanism, in which enhanced toxic gain-of-function of polyglutamine-expanded ATXN1 leads to neurodegeneration, while a simultaneous loss-of-function of other stable endogenous protein complexes, Atxn1-Cic, contributes to the SCA1 phenotypes (Figure 6B).

It was previously reported that reduction of normal functions of genes involved in other polyglutamine diseases results in enhanced pathology, providing evidence for concomitant gain- and loss-of-function mechanisms in polyglutamine disorders [39]–[43]. In SBMA transgenic mouse models expressing polyglutamine-expanded androgen receptor (AR), loss of endogenous AR protein resulted in accelerated motor neuron degeneration [39]. These studies suggested two independent pathways contributing to SBMA pathogenesis: gain-of-function due to mutant AR nuclear toxicity and loss of AR trophic effects on motor neurons [39]. Conditional deletion of *Htt* in mouse forebrain leads to several features reminiscent of Huntington disease, including motor deficits, tremors and progressive degeneration of the striatum and cortex, hinting that loss of htt function could contribute to these phenotypes in HD [42]. Moreover, loss of wild-type Htt function leads to enhanced neurodegeneration in transgenic models expressing polyglutamine-expanded Htt, while overexpression of wild-type Htt reduces toxicity caused by mutant htt [40]–[43]. The mechanism by which loss of Htt contributes to HD is unclear at this time; it might involve either its anti-apoptotic properties, its role in BDNF-mediated neuroprotection, both, or some other yet to be determined function [44]–[47]. Our studies comparing *Atxn1* ^−/−^ and SCA1 knock-in mice pinpoint Cic-dependent transcriptional repression as one of the molecular pathways mediating the partial loss-of-function component in SCA1 pathogenesis.

Loss of normal endogenous function of mutant proteins may also play a role in other dominant neurodegenerative diseases caused by gain-of-function mutations. The Parkinson's disease model mice overexpressing the mutant A53T *SNCA* gene lacking endogenous alpha-synuclein, exhibit worsened synucleinopathy when compared to littermates carrying wild-type *Snca* alleles [48]. In Alzheimer disease (AD), increased aggregation of the amyloid beta peptides induced by AD-related presenilin mutations is thought to be a consequence of a dominant gain-of-function mechanism [49]. However, loss of function of presenilin in the mouse brain results in phenotypes strikingly reminiscent of AD (progressive memory loss and neurodegeneration) in the absence of beta-amyloid deposition [50], [51]. These results suggest that altered pathways leading to Alzheimer disease can be caused from a combination of dominant gain of function and/or loss of function mechanisms. The potential prevalence of mutations that lead to both loss- and gain-of-function in human neurological diseases underscores the importance of understanding the endogenous functions of causative genes through the careful analysis of loss-of-function models, which may uncover critical pathways leading to pathogenesis.

## Materials and Methods

### Mouse lines

All mouse lines used in this study have been previously described [9], [13], [24]. *Atxn1^154Q^* ^/+^ and *Atxn1* ^−/−^ mice have been backcrossed into the C75Bl/6J strain for over ten generations. In the case of *Atxn1L duplication* (*Atxn1L^dp^*) mice, all experiments described in this study were done in mice backcrossed for at least 7 generations into the C75Bl/6J strain. *Atxn1* ^−/−^ mice were bred to *Atxn1L^dp^* ^/+^ mice, and *Atxn1* ^−/−^ mice carrying the *Atxn1L duplication* locus were generated by intercrossing the progeny. Mouse experiments followed protocols approved by the Baylor College of Medicine Institutional Animal Care and Use Committee (IACUC).

### RNA isolation

Total RNA isolation from adult mouse cerebella was performed as described elsewhere [52]. Briefly, RNA was extracted from cerebella of 16-week-old mice using TRIzol reagent (Invitrogen Corporation, Carlsbad, CA), DNaseI-treated, and purified using the RNeasy mini kit according to the manufacturer's protocol (Qiagen, Valencia, CA).

### Microarray analysis with exon arrays

We used the Affymetrix Mouse Exon 1.0 ST microarray, which carries 1.2 million probe-sets covering one million exon clusters, with an average of 40 probes per gene. The exon array data were analyzed as previously described [52]. Briefly, raw data were processed in the R statistical programming environment using locally developed methods and the exonmap package. RMA normalization was applied, and linear models were calculated to analyze genotype effects for each gene. Genomic annotations were obtained from UCSC (http://genome.ucsc.edu). The normalized probe level data were then averaged within each exon to produce exon-level data for each gene for each animal. A two-way ANOVA with main effects for genotype and exonic region was calculated for each gene. The ANOVA model was fit using weighted least squares analysis where the weights were determined according to the probe counts within each exon. Since separate wild-type (WT) control littermates were used in the *Atxn1^−/−^* and *Atxn1^154Q/+^* experiments, a separate linear model was estimated for each gene in each model (one fit for each WT background). A linear contrast was calculated comparing the WT and mutant cross-exon means for each gene. The cut-off rule for determining genes was a fold change threshold of +/−0.1 in both experiments, and a linear step up false discovery rate (FDR) of less than 0.01 value for the T-statistic corresponding to the linear contrast comparing each WT strain with its corresponding mutant. The gene set determined by this fold change and FDR multiplicity corrected cutoff, corresponds to a median raw marginal p-value of less than 0.00015 for the underlying T-statistics.

We performed Gene Ontology (GO) analysis on the obtained data using locally developed software and methods [52]. Briefly, the gene ontology vocabulary and current mouse annotations were obtained from the GO website (9/1/2007 build). The mouse exon array was mapped to Entrez identifiers, and these identifiers were mapped to the GO data structure using the available annotations. Using our local ontology analysis system (OntologyTraverser), we tabulated the genes annotated at or below each GO node for the entire exon array. We then used a hypergeometric sampling model to examine the statistical representation of each GO node for genes in our gene sets. In order to make comparisons between sets, we took differences between the standardized scores determined for each gene set. Because of the extreme overlapping structure of the GO, many GO nodes report duplicate or redundant information. To avoid this problem, we calculated the GO covariance structure and used this estimate to compute de-correlated GO scores. For the KEGG pathway analysis, the overlapping gene list containing the 197 most significant genes was uploaded and analyzed using the web-based Functional Annotation Bioinformatics Microarray Analysis DAVID 6.7 (National Institute of Allergy and Infectious Diseases NIH, david.abcc.ncifcrf.gov/).

### Real-time quantitative reverse transcriptase-polymerase chain reaction (qRT–PCR)

For the independent validation of the exon array data, real time qRT-PCR assays were performed on more than eight mice of each genotype. cDNA was synthesized from 1 µg of RNA using the RT2 First Strand Kit (SuperArray Bioscience Corporation, Frederick, MD). Quantitative real-time PCR reactions were performed on 10 ng of cDNA using RT2 SYBR Green/ROX PCR master mix and commercially available primers (SuperArray Bioscience Corporation, Frederick, MD). All RNA samples were analyzed in triplicate and normalized relative to *Gapdh* levels.

For validation of *Rorα* targets, cDNA was synthesized from RNA isolated from seven *Atxn1* ^−/−^ mice and control littermates, using Superscript III (Invitrogen). Quantitative real-time PCR reactions were performed on 10 ng of cDNA using the SYBR Green PCR Master Mix (Applied Biosystems) using the following primer sets:

*Itpr1* fw 5′- GGGCCAACAGCACTACAGATG-3′

 rv 5′-CTTCTTTTCCAAGTCTGCAGCAT-3′

*Grid2* fw 5′- CCCGCATTGAGAGCTCCAT-3′

 rv 5′- GCCATAAGGGATATCTGTTTGCTT-3′

*Inpp5a* fw 5′- TGGTCAAGAAAAGGCTTCATCA-3′

 rv 5′- CCAAGTCGAAGGCACAGTCA-3′

*Grm1* fw 5′- TCCTCTGACCTGAGACCAATAGC-3′

 rv 5′- CGCGTTAGTGGCCATAAGCT-3′

### Chromatin Immunoprecipitation-polymerase chain reaction analysis (ChIP-PCR)

ChIP was performed as previously described [52]. Cerebella were dissected from mice of each genotype at 7–8 weeks of age and incubated in 1% formaldehyde for 10 minutes at room temperature to cross-link DNA to associated proteins. Chromatin was treated with micrococcal nuclease and sheared by sonication to generate fragments with an average length of ∼100–200 bps, as determined by agarose gel electrophoresis. For immunoprecipitation, 200 µl of chromatin was diluted 1∶10 in ChIP dilution buffer (Millipore Corporation, Billerica, MA) and 1% of the diluted sample was saved as input. The sample was first precleared with protein A Dynabeads (Invitrogen Corporation, Carlsbad, CA), then incubated overnight with protein A Dynabeads that were pre-blocked with salmon sperm DNA and coupled to 20 µl of affinity purified rabbit anti-Atxn1 (11NQ) antibody, or 4 µl of guinea pig polyclonal anti-Cic antibody. Mock immunoprecipitations using nonspecific preimmune sera for each antibody were included as negative controls. After immunoprecipitation, the beads were washed at room temperature with low salt buffer, followed by high salt buffer, LiCl buffer (Millipore Corporation, Billerica, MA), and TE buffer (10 mM Tris-HCl pH 7.4, 1 mM EDTA pH 8.0). Elution was performed twice in 250 µl of fresh elution buffer (1% SDS, 0.1 M NaHCO_3_) for 15 minutes at room temperature. The eluates were combined, the crosslinks were reversed, and DNA was purified with Qiagen PCR cleanup kit (Qiagen, Valencia, CA) and recovered in 30 µl of 10 mM Tris-HCl pH 8.0. One or two µl of DNA were used for each PCR reaction using primer pairs for the promoter regions of the following genes:

*Etv5* fw *5′*-GGGGAAGCTTAGCTGAGTCAGTGAA-3′

 rv 5′- GTTTCTGTGTGTGGAATGACGAATTC-3′

*Ccnd1* fw 5′-GGTTAACTGAATGGACTCCTAAGTTT-3′

 rv 5′-GGAAATGTGTGTGAATAGTTCGCCTA-3′

Capicua binding to the promoters was also analyzed using ChIP followed by quantitative real-time PCR (SYBR green). Quantitative real-time PCR experiments were performed in triplicate on three independent sets of samples. Relative amounts of immunoprecipitated DNA were determined based on the threshold cycle (Ct) value for each PCR reaction. In order to control for variation between ChIP fractions, for every gene promoter studied, a ΔCt value was calculated for each sample (*Atxn1* ^+/−^, *Atxn1^154Q^* ^/+^, *Atxn1* ^−/−^) by subtracting the Ct value for the input (Ct_Input_) from the Ct value for the immunoprecipitated sample (Ct_antibody_ or Ct_preimmune_). Since the input DNA fraction represents only 1% of the total material, the Ct_Input_ value was first adjusted for this dilution factor by subtracting 6.644 cycles (Log_2_ of 100), then substracted from the immunoprecipitated samples using the following formula:
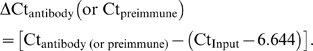
Differences between the specific immunoprecipitation and the preimmune serum background were then determined and plotted as fold enrichment over the preimmune serum (for each genotype sample: ΔCt_antibody_/ΔCt_preimmune_).

Primer and sequences for the promoter regions used were as follows:

*Etv5 qA* (highly conserved; two predicted Cic-binding sites):

 *fw* *5′*- TTGCTCCTGATCACACATGC -3′

 rv 5′- GCTGGAACCTCGTGAATGAT -3′

*Etv5 qB* (conserved; ∼1 Kb upstream of positive region Etv5 qA)

 *fw* *5*′- AATCAGCACCGGCTTGTTTA-3′

 rv 5′- CTAAGCTTCCCCCTCAGGTC-3′

*Ccnd1 qA* (highly conserved; ∼150 bp upstream of predicted Cic-binding site)

 fw 5′- AAATTTGCATGAGCCAATCC-3′

 rv 5′- GCAGAGCTCAACGAAGTTCC-3′

*Ccnd1 qB* (poorly conserved; ∼400bp downstream of predicted Cic-binding site)

 fw 5′- GGTCGTGGTTAACTGAATGGA -3′

 rv 5′- AGGTGGTGGAACCGCTTTAT-3′

### Co-immunoprecipitation studies

Cerebella from three mice of each genotype were dissected, and dounce-homogenized in 1 ml of ice-cold TST buffer (10 mM Tris–HCl, pH 7.5, 0.9% NaCl, 0.05% Tween 20). Protein was adjusted to 0.5 mg in 200 µl with TST buffer (10 mM Tris–HCl, pH 7.5, 0.9% NaCl, 0.05% Tween 20) and then added 800 ml cold PBS. Fifteen microlitters of the extract were saved as input. The extract was pre-cleared using protein A Sepharose beads. BSA-blocked protein A Sepharose beads were coupled to 4 µl of polyclonal guinea pig anti-Cic serum or pre-immune serum for 1 hr at room temperature, and the diluted extract was incubated with the antibody-coupled beads overnight at 4°C. Four washes were carried out using ice-cold TST buffer and the pellet was resuspended in sample buffer. Input and pellets were analyzed by SDS-PAGE and protein blot.

### Luciferase assays

Luciferase reporter assays for Cic-dependent repression were performed as previously described [18]. HEK293T cells in 24-well plates were co-transfected using Lipofectamine 2000 (Invitrogen) with 50 ng of the pGL3-Promoter (Promega) containing six copies of CIC binding sites (TGAATGAA or TGAATGGA), 10 ng of pRL-TK, and 10 ng of expression plasmids for Atxn1[2Q] (wild-type), Atxn1L and Cic-expressing plasmids as indicated. All constructs have also been previously described [18], [24]. The total amount of DNA transfected was kept constant by adding pcDNA3.1(-) (Invitrogen). Luciferase activities were measured using the dual luciferase reporter assay system (Promega).

### Contextual fear conditioning

For this task, the mice were trained in a novel environment with a neutral stimulus (a tone) paired with a foot shock. The mice are placed in a different context with the same tone (cued test) or back in the same environment without a tone (contextual test) 24 h later. Mice of each genotype were placed in a Med Associates/Actimetrics chamber system where a 30 second tone was followed by a 2 second foot shock at 1.5 mA. The tone–foot shock were repeated at 2 min. Twenty-four hours later, the mice were tested for freezing in the same chamber with no tone to evaluate contextual fear. Scoring for freezing is automated in this system. Analysis was performed using ANOVA and t-test analysis.

### Dowel and wire hang paradigms

Mice were placed on the center of a 0.9 cm wooden dowel suspended between two platforms. If the mouse walked off of the dowel onto the platform, the mouse was placed back in the center. The test lasted 2 min beginning when the mouse was placed on the dowel. Data were analyzed using ANOVA and Student's t-test.

## Supporting Information

Figure S1 Cellular component gene ontology for genes commonly up-regulated in *Atxn1* ^−/−^ and *Atxn1^154Q^* ^/+^ cerebella. Gene ontology categories shown were significantly enriched (positive z score, green) or depleted (negative z score, green) with the de-correlated z score for enrichment plotted in the x-axis. Only gene ontology categories with more than one gene represented and a z score>|+/−2| are represented.
(0.24 MB TIF)

Figure S2 Biological process gene ontology for genes commonly up-regulated in *Atxn1* ^−/−^ and *Atxn1^154Q^* ^/+^ cerebella. Gene ontology categories shown were significantly enriched (positive z score, green) or depleted (negative z score, green) with the de-correlated z score for enrichment plotted in the x-axis. Only gene ontology categories with more than one gene represented and a z score>|+/−2| are represented.
(0.53 MB TIF)

Figure S3 Molecular function gene ontology for genes commonly up-regulated in *Atxn1* ^−/−^and *Atxn1^154Q^* ^/+^ cerebella. Gene ontology categories shown were significantly enriched (positive z score, green) or depleted (negative z score, green) with the de-correlated z score for enrichment plotted in the x-axis. Only gene ontology categories with more than one gene represented and a z score>|+/−2| are represented.
(0.51 MB TIF)

Figure S4 Cellular component gene ontology for genes commonly down-regulated in *Atxn1* ^−/−^ and *Atxn1^154Q^* ^/+^ cerebella. Gene ontology categories shown were significantly enriched (positive z score, green) or depleted (negative z score, green) with the de-correlated z score for enrichment plotted in the x-axis. Only gene ontology categories with more than one gene represented and a z score>|+/−2| are represented.
(0.38 MB TIF)

Figure S5 Biological process gene ontology for genes commonly down-regulated in *Atxn1* ^−/−^ and *Atxn1^154Q^* ^/+^ cerebella. Gene ontology categories shown were significantly enriched (positive z score, green) or depleted (negative z score, green) with the de-correlated z score for enrichment plotted in the x-axis. Only gene ontology categories with more than one gene represented and a z score>|+/−2| are represented.
(0.74 MB TIF)

Figure S6 Molecular function gene ontology for genes commonly down-regulated in *Atxn1* ^−/−^ and *Atxn1^154Q^* ^/+^ cerebella. Gene ontology categories shown were significantly enriched (positive z score, green) or depleted (negative z score, green) with the de-correlated z score for enrichment plotted in the x-axis. Only gene ontology categories with more than one gene represented and a z score>|+/−2| are represented.
(0.73 MB TIF)

Figure S7 Real time qRT-PCR validation of Rorα targets in *Atxn1^−/−^* cerebella. Four out of six tested genes were significantly down-regulated in *Atxn1^−/−^* cerebella. Error bars in graph represent +/− SEM *p<0.05.
(0.30 MB TIF)

Figure S8 Open Field Analysis to measure activity in pure C57Bl/6J *Atxn^−/−^* mice. (A) *Atxn1* ^−/−^ mice at 10–11 weeks of age spend more time in the center of the field compared to wild-type littermates, suggesting they are less anxious. (B) *Atxn1* ^−/−^ mice and wild-type littermates traveled similar total distances. (C) *Atxn1* ^−/−^ mice had decreased rearing, as measured by vertical activity in the open field. Since *Atxn1* ^−/−^ mice show increased center/total distance, their reduced vertical activity probably reflects motor defects affecting rearing, and not increased anxiety. Error bars represent +/− SEM, **p<0.005.
(0.31 MB TIF)

Figure S9 Light/Dark box and Elevated Plus maze to test for anxiety in pure C57Bl/6J *Atxn1^−/−^* mice. *Atxn1* ^−/−^ mice (n = 10) and wild-type controls (n = 9) were tested at 12 weeks of age (A) *Atxn1* ^−/−^ mice had a small trend to spend more time in the light side than wild-type controls, although this trend does not reach significance (p = 0.26). (B) Both wild type and *Atxn1* ^−/−^ mice make the same number of transitions between the light and dark side of the box. (C) In the elevated plus maze, *Atxn1* ^−/−^ mice spent more time in the open arms than controls, and (D) also made more arm entries than controls. This shows that pure C57Bl/6J *Atxn1* ^−/−^ mice are not hypoactive, as reported before for *Atxn1* ^−/−^ mice in a mixed C57Bl/6J/129svEv background. Error bars represent +/− SEM, ***p<0.00001.
(0.44 MB TIF)

Figure S10 Conditioned Fear Analysis to measure Pavlovian learning in *Atxn1^−/−^* mice. Seven mice of each genotype were tested at 12 weeks of age. (A) In the contextual conditioned fear test, mice were exposed to a tone paired to a foot shock and 24 hrs later, placed in the same chamber and the amount of freezing behavior is recorded, (B) In the cued test, only the tone is administered in a different chamber and freezing is recorded. *Atxn1* ^−/−^ mice show less freezing both in the contextual and cued test (A and B), indicating amygdala and hippocampal deficits in learning and memory. Error bars represent +/− SEM, *p<0.05,***p<0.001.
(0.27 MB TIF)

Figure S11 Dowel and wire hang analysis to measure gross motor ability in *Atxn1^−/−^* mice. In the Dowel test for motor coordination, mice are placed on a 0.9 cm rod and the time that it takes to reach the side and number of side touches in 2 minutes is recorded. *Atxn1* ^−/−^mice (n = 11) and wild-type littermates (n = 9) were tested at 12 weeks. (A) *Atxn1* ^−/−^ mice performed poorly in the dowel, with increased latency for to reach the sides for the first time. (B) *Atxn1* ^−/−^mice also made less number of side touches in 2 min. (C and D) The wire hang test is similar to the dowel, except a wire is used instead of a rod. *Atxn1* ^−/−^ mice had a trend to reach the sides fewer times in 2 minutes than the controls (D), albeit not significant (p<0.06) Error bars +/− SEM, *p<0.05,**p<0.01.
(0.52 MB TIF)

Table S1 List of 197 commonly altered genes in Atxn1^−/−^ and Atxn1^154Q/+^ cerebella. (p<0.01 Fold Change |+/−0.1| log_2_ scale).
(0.37 MB DOC)

Table S2 KEGG pathway analysis of the 197 commonly altered genes in *Atxn1* ^−/−^ and *Atxn1^154Q^* ^/+^ cerebella.
(0.06 MB DOC)
