# Supplementary figures and images for: Partial Loss of Ataxin-1 Function Contributes to Transcriptional Dysregulation in Spinocerebellar Ataxia Type 1 Pathogenesis
Source: PLoS Genet. 2010 Jul 8;6(7):e1001021. doi: 10.1371/journal.pgen.1001021 (PMC2900305; doi:10.1371/journal.pgen.1001021)

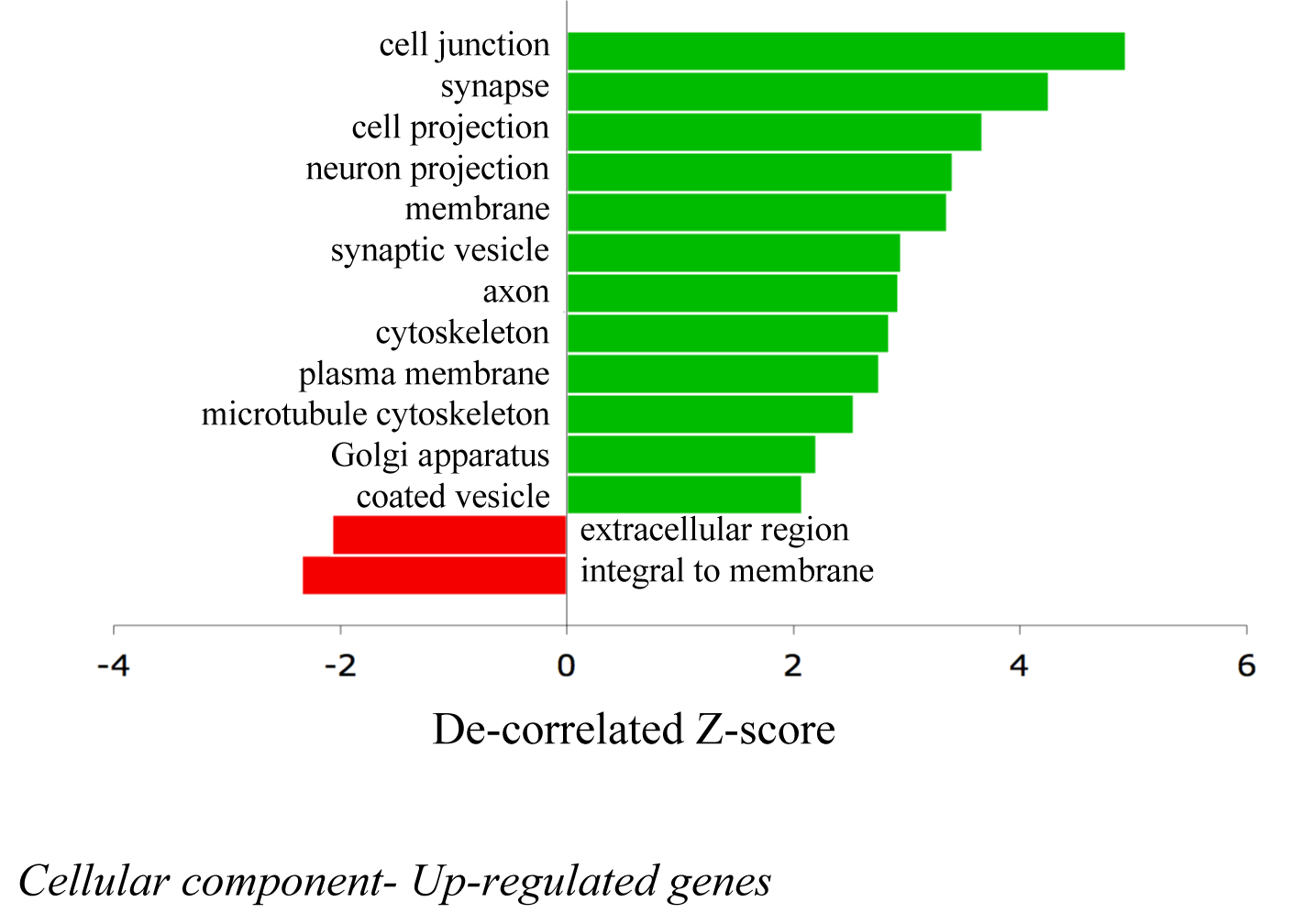

Supplement: Figure S1 — Cellular component gene ontology for genes commonly up-regulated in Atxn1 −/− and Atxn1154Q /+ cerebella. Gene ontology categories shown were significantly enriched (positive z score, green) or depleted (negative z score, green) with the de-correlated z score for enrichment plotted in the x-axis. Only gene ontology categories with more than one gene represented and a z score>|+/−2| are represented. (0.24 MB TIF) [file pgen.1001021.s001.tif]

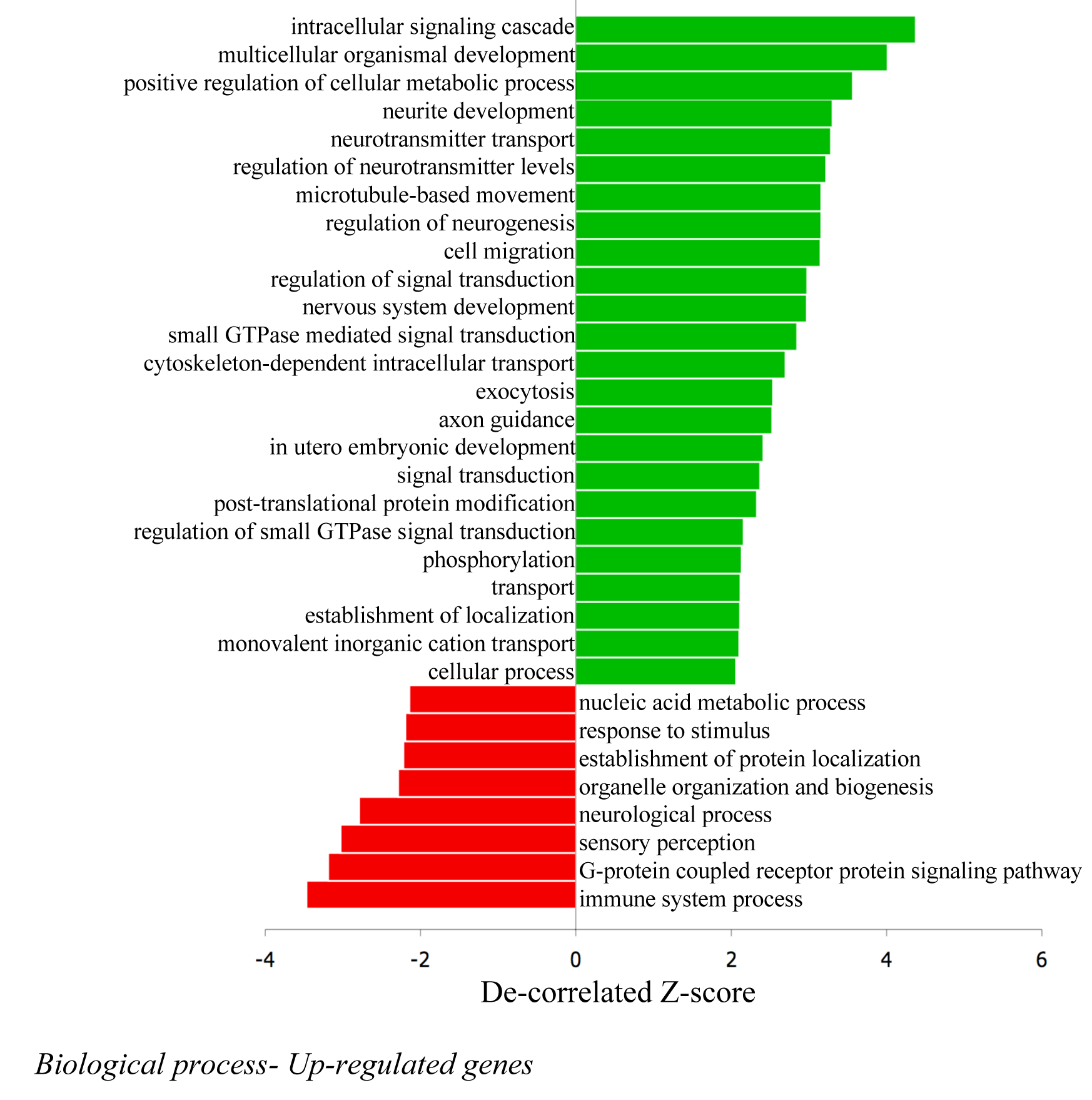

Supplement: Figure S2 — Biological process gene ontology for genes commonly up-regulated in Atxn1 −/− and Atxn1154Q /+ cerebella. Gene ontology categories shown were significantly enriched (positive z score, green) or depleted (negative z score, green) with the de-correlated z score for enrichment plotted in the x-axis. Only gene ontology categories with more than one gene represented and a z score>|+/−2| are represented. (0.53 MB TIF) [file pgen.1001021.s002.tif]

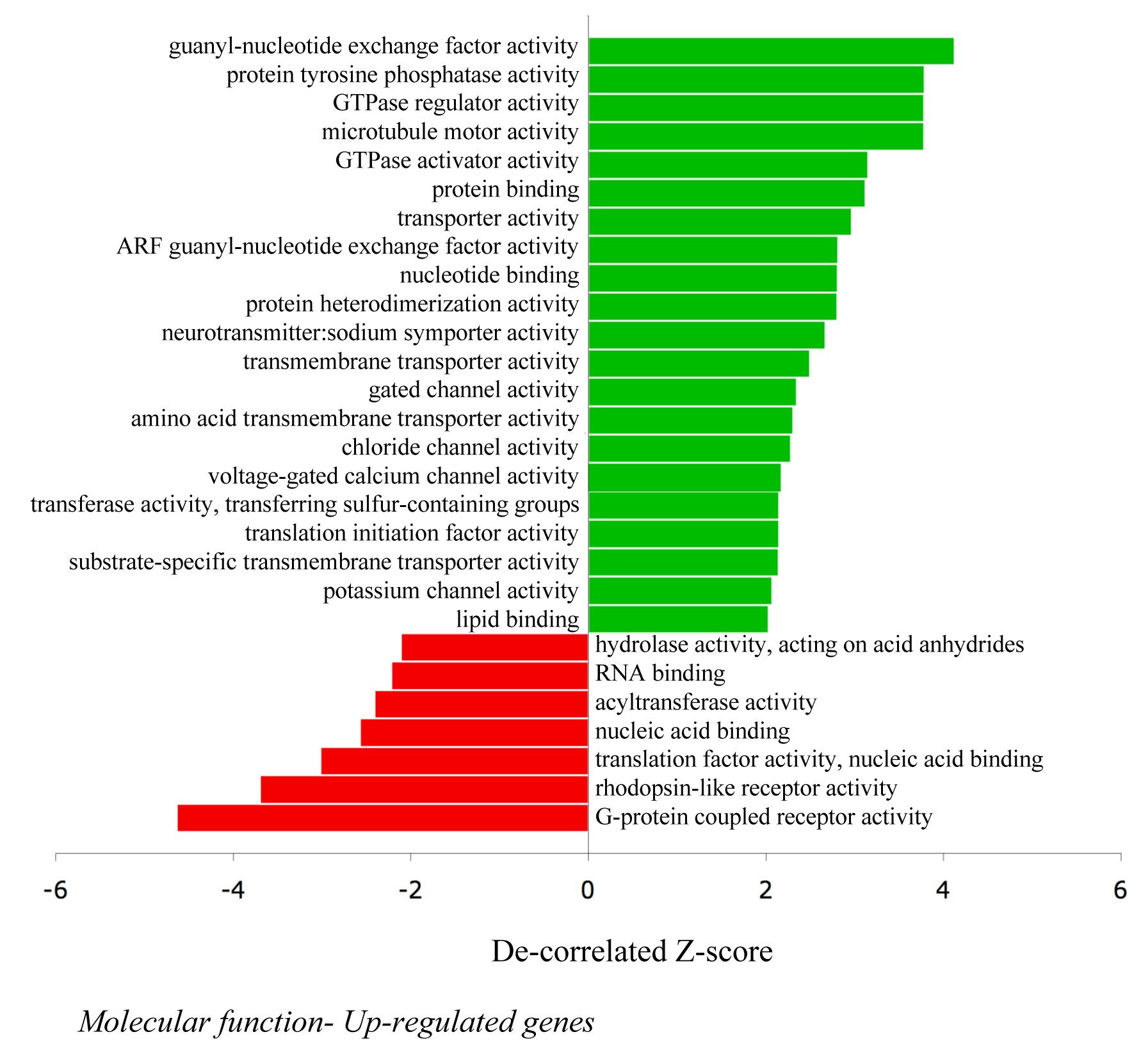

Supplement: Figure S3 — Molecular function gene ontology for genes commonly up-regulated in Atxn1 −/−and Atxn1154Q /+ cerebella. Gene ontology categories shown were significantly enriched (positive z score, green) or depleted (negative z score, green) with the de-correlated z score for enrichment plotted in the x-axis. Only gene ontology categories with more than one gene represented and a z score>|+/−2| are represented. (0.51 MB TIF) [file pgen.1001021.s003.tif]

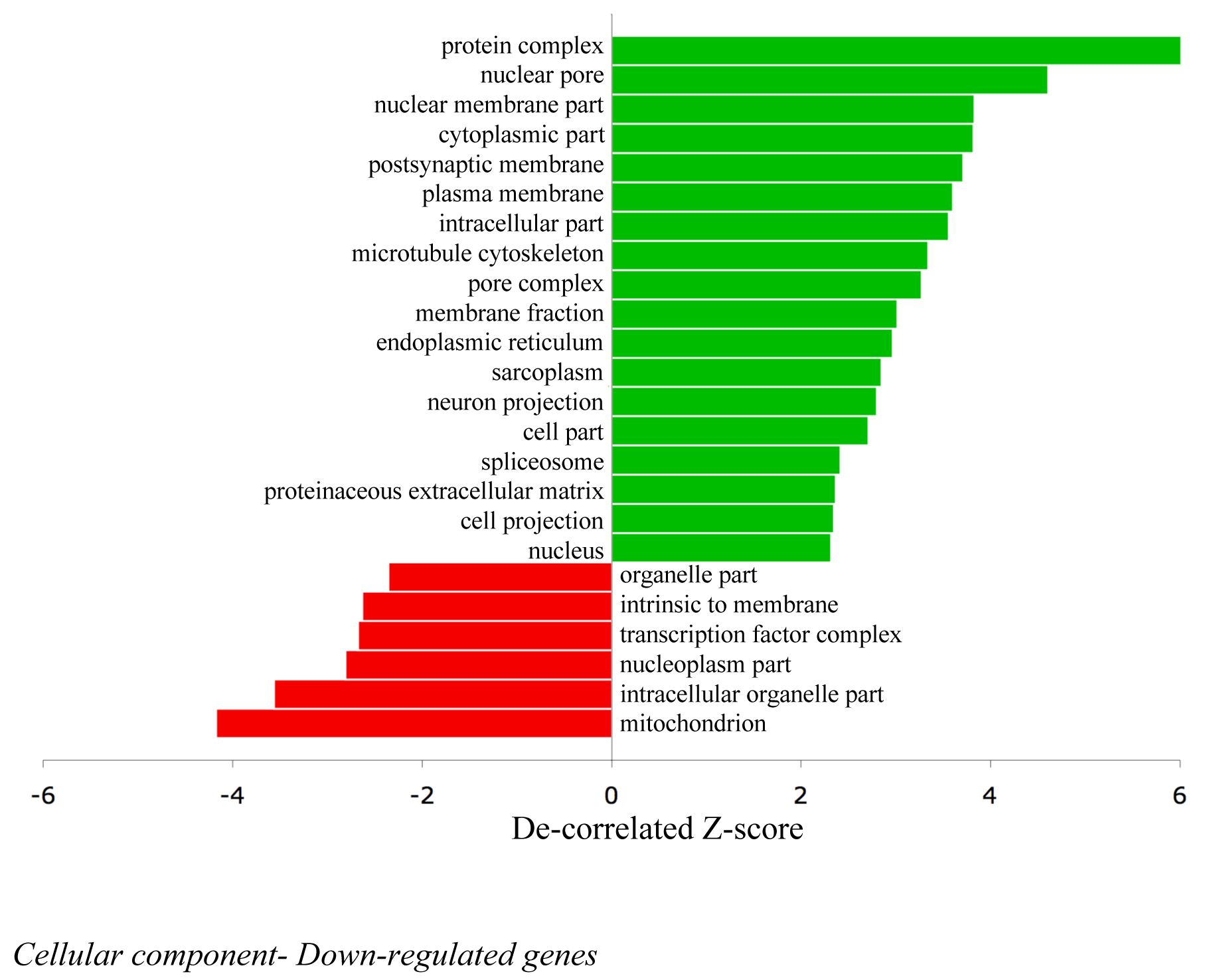

Supplement: Figure S4 — Cellular component gene ontology for genes commonly down-regulated in Atxn1 −/− and Atxn1154Q /+ cerebella. Gene ontology categories shown were significantly enriched (positive z score, green) or depleted (negative z score, green) with the de-correlated z score for enrichment plotted in the x-axis. Only gene ontology categories with more than one gene represented and a z score>|+/−2| are represented. (0.38 MB TIF) [file pgen.1001021.s004.tif]

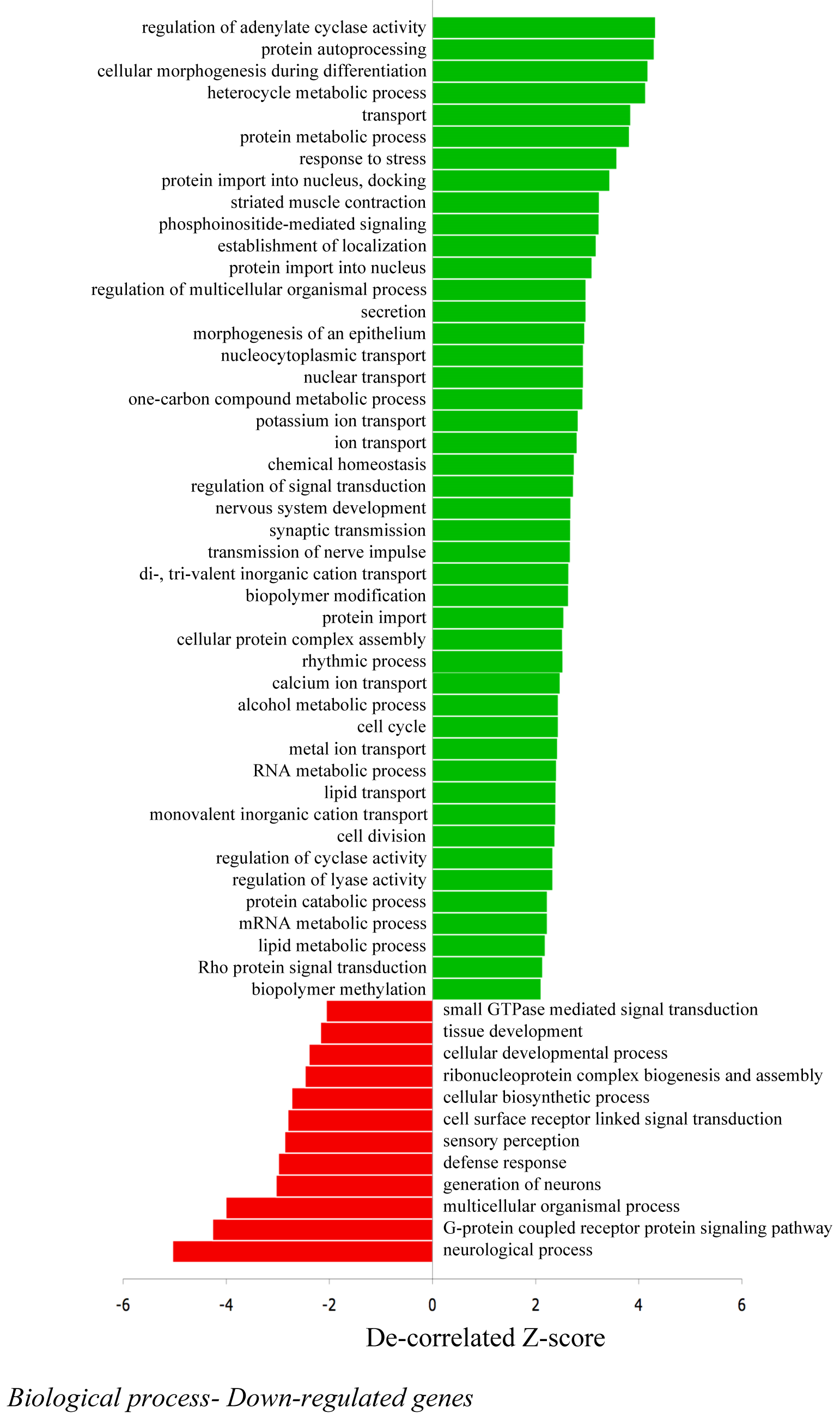

Supplement: Figure S5 — Biological process gene ontology for genes commonly down-regulated in Atxn1 −/− and Atxn1154Q /+ cerebella. Gene ontology categories shown were significantly enriched (positive z score, green) or depleted (negative z score, green) with the de-correlated z score for enrichment plotted in the x-axis. Only gene ontology categories with more than one gene represented and a z score>|+/−2| are represented. (0.74 MB TIF) [file pgen.1001021.s005.tif]

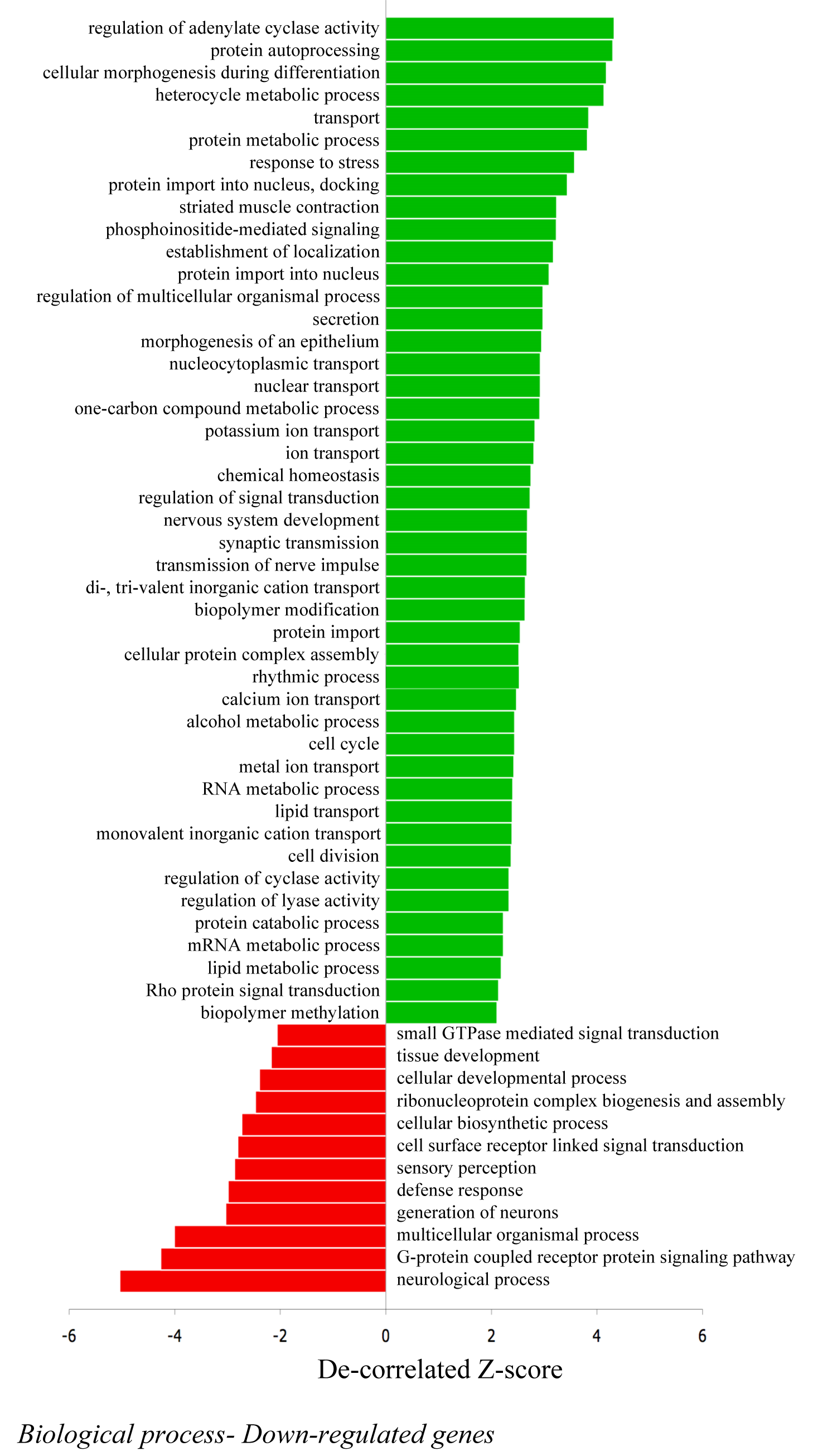

Supplement: Figure S6 — Molecular function gene ontology for genes commonly down-regulated in Atxn1 −/− and Atxn1154Q /+ cerebella. Gene ontology categories shown were significantly enriched (positive z score, green) or depleted (negative z score, green) with the de-correlated z score for enrichment plotted in the x-axis. Only gene ontology categories with more than one gene represented and a z score>|+/−2| are represented. (0.73 MB TIF) [file pgen.1001021.s006.tif]

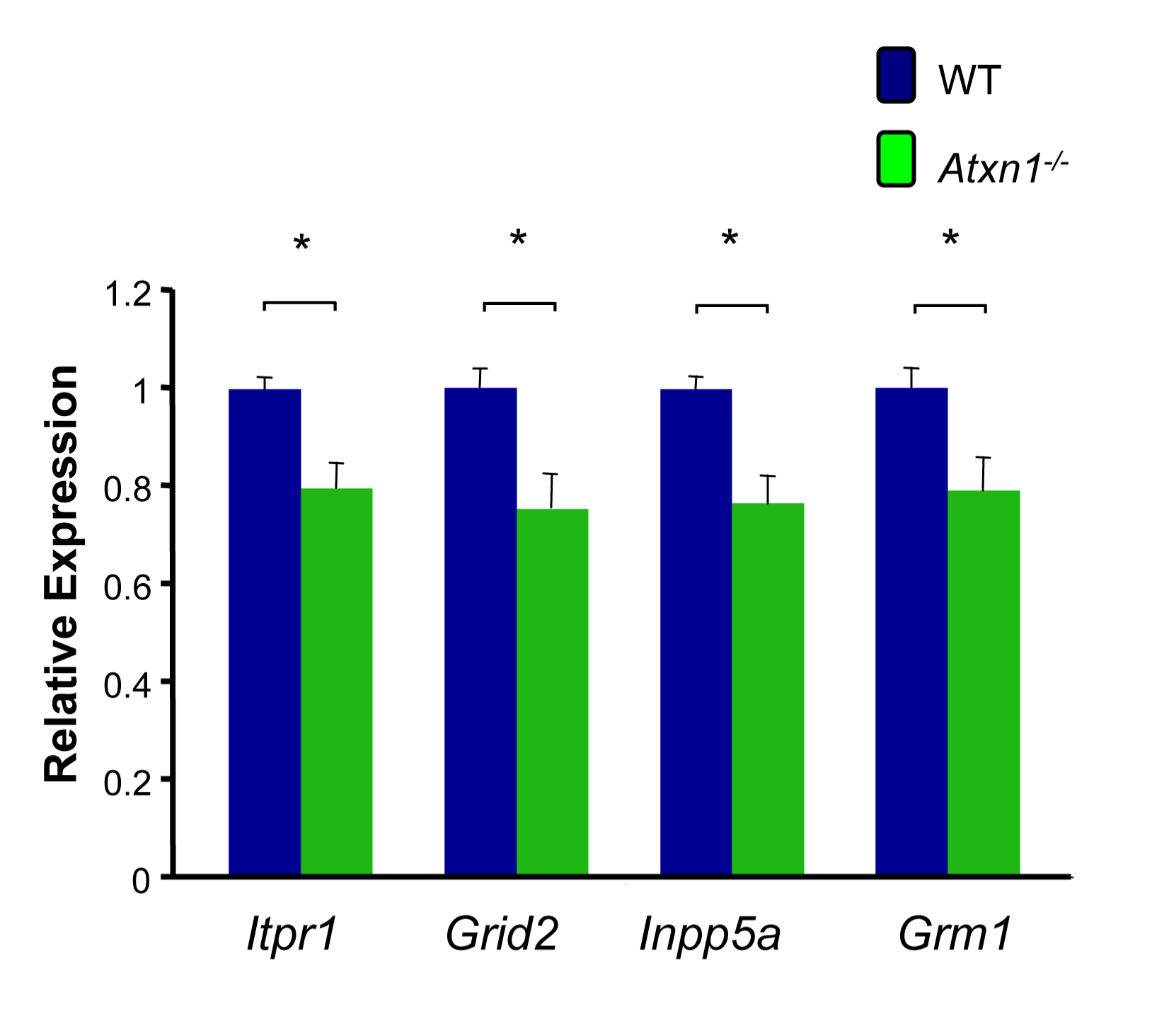

Supplement: Figure S7 — Real time qRT-PCR validation of Rorα targets in Atxn1−/− cerebella. Four out of six tested genes were significantly down-regulated in Atxn1−/− cerebella. Error bars in graph represent +/− SEM *p<0.05. (0.30 MB TIF) [file pgen.1001021.s007.tif]

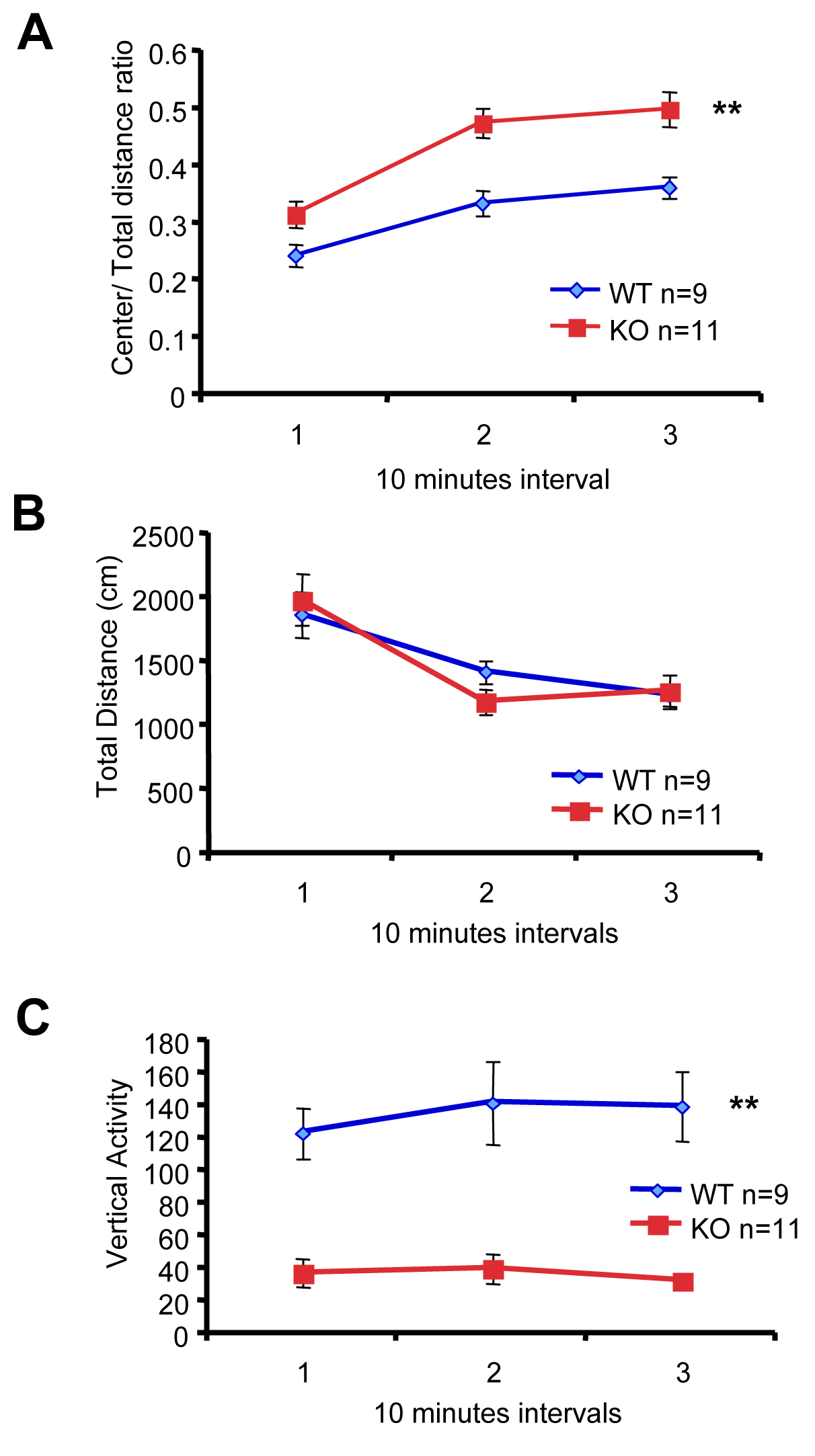

Supplement: Figure S8 — Open Field Analysis to measure activity in pure C57Bl/6J Atxn−/− mice. (A) Atxn1 −/− mice at 10–11 weeks of age spend more time in the center of the field compared to wild-type littermates, suggesting they are less anxious. (B) Atxn1 −/− mice and wild-type littermates traveled similar total distances. (C) Atxn1 −/− mice had decreased rearing, as measured by vertical activity in the open field. Since Atxn1 −/− mice show increased center/total distance, their reduced vertical activity probably reflects motor defects affecting rearing, and not increased anxiety. Error bars represent +/− SEM, **p<0.005. (0.31 MB TIF) [file pgen.1001021.s008.tif]

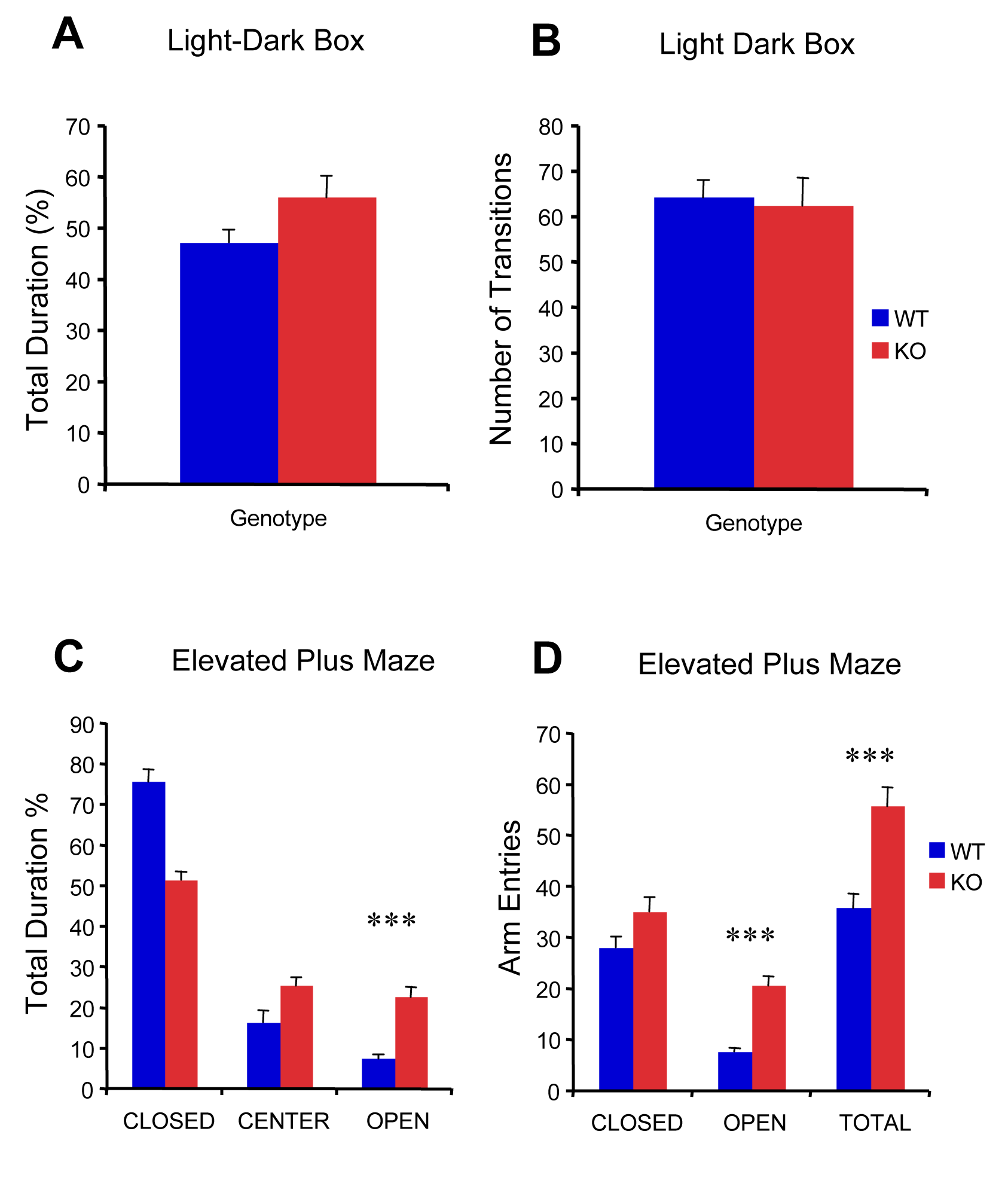

Supplement: Figure S9 — Light/Dark box and Elevated Plus maze to test for anxiety in pure C57Bl/6J Atxn1−/− mice. Atxn1 −/− mice (n = 10) and wild-type controls (n = 9) were tested at 12 weeks of age (A) Atxn1 −/− mice had a small trend to spend more time in the light side than wild-type controls, although this trend does not reach significance (p = 0.26). (B) Both wild type and Atxn1 −/− mice make the same number of transitions between the light and dark side of the box. (C) In the elevated plus maze, Atxn1 −/− mice spent more time in the open arms than controls, and (D) also made more arm entries than controls. This shows that pure C57Bl/6J Atxn1 −/− mice are not hypoactive, as reported before for Atxn1 −/− mice in a mixed C57Bl/6J/129svEv background. Error bars represent +/− SEM, ***p<0.00001. (0.44 MB TIF) [file pgen.1001021.s009.tif]

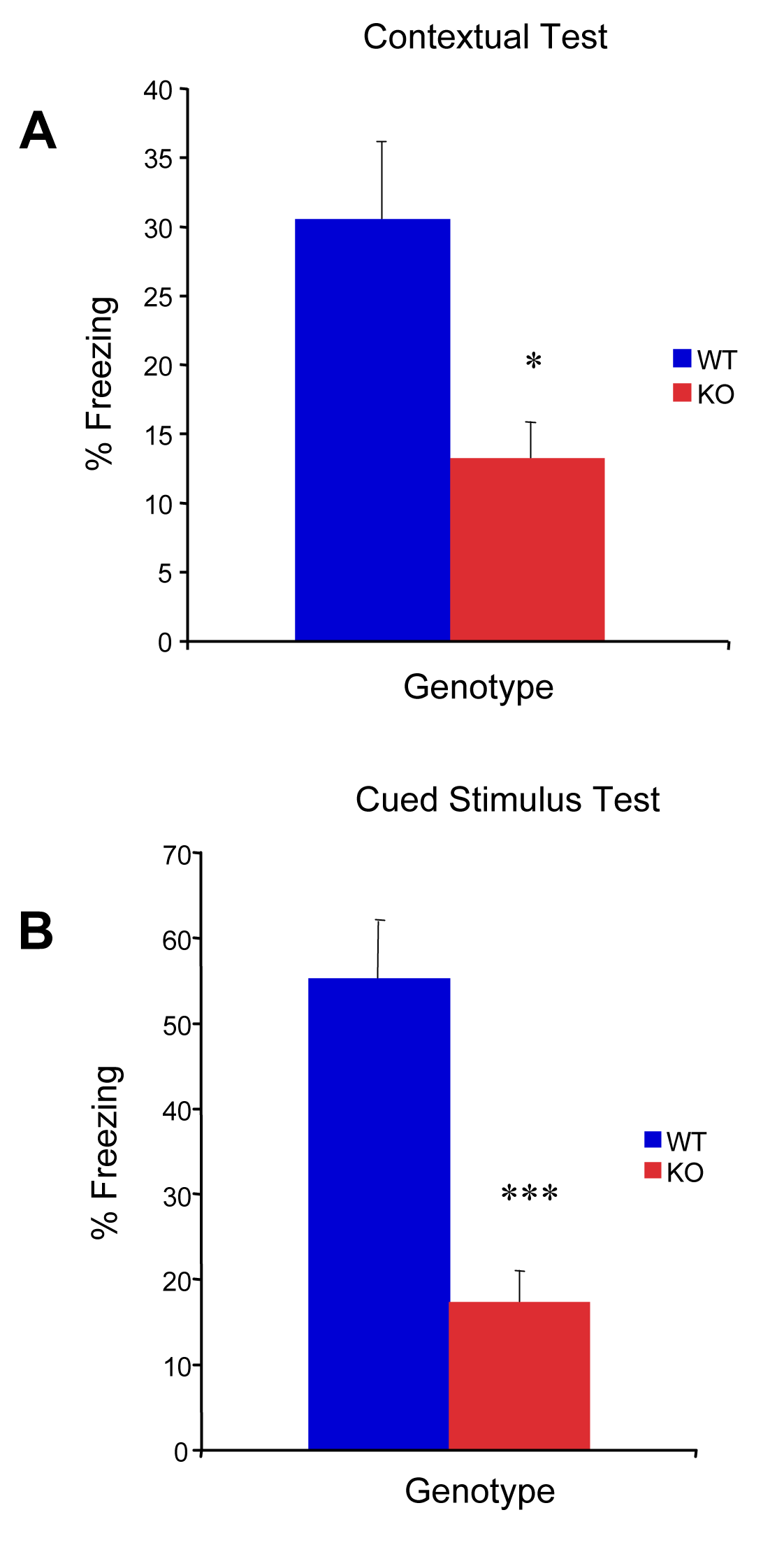

Supplement: Figure S10 — Conditioned Fear Analysis to measure Pavlovian learning in Atxn1−/− mice. Seven mice of each genotype were tested at 12 weeks of age. (A) In the contextual conditioned fear test, mice were exposed to a tone paired to a foot shock and 24 hrs later, placed in the same chamber and the amount of freezing behavior is recorded, (B) In the cued test, only the tone is administered in a different chamber and freezing is recorded. Atxn1 −/− mice show less freezing both in the contextual and cued test (A and B), indicating amygdala and hippocampal deficits in learning and memory. Error bars represent +/− SEM, *p<0.05,***p<0.001. (0.27 MB TIF) [file pgen.1001021.s010.tif]

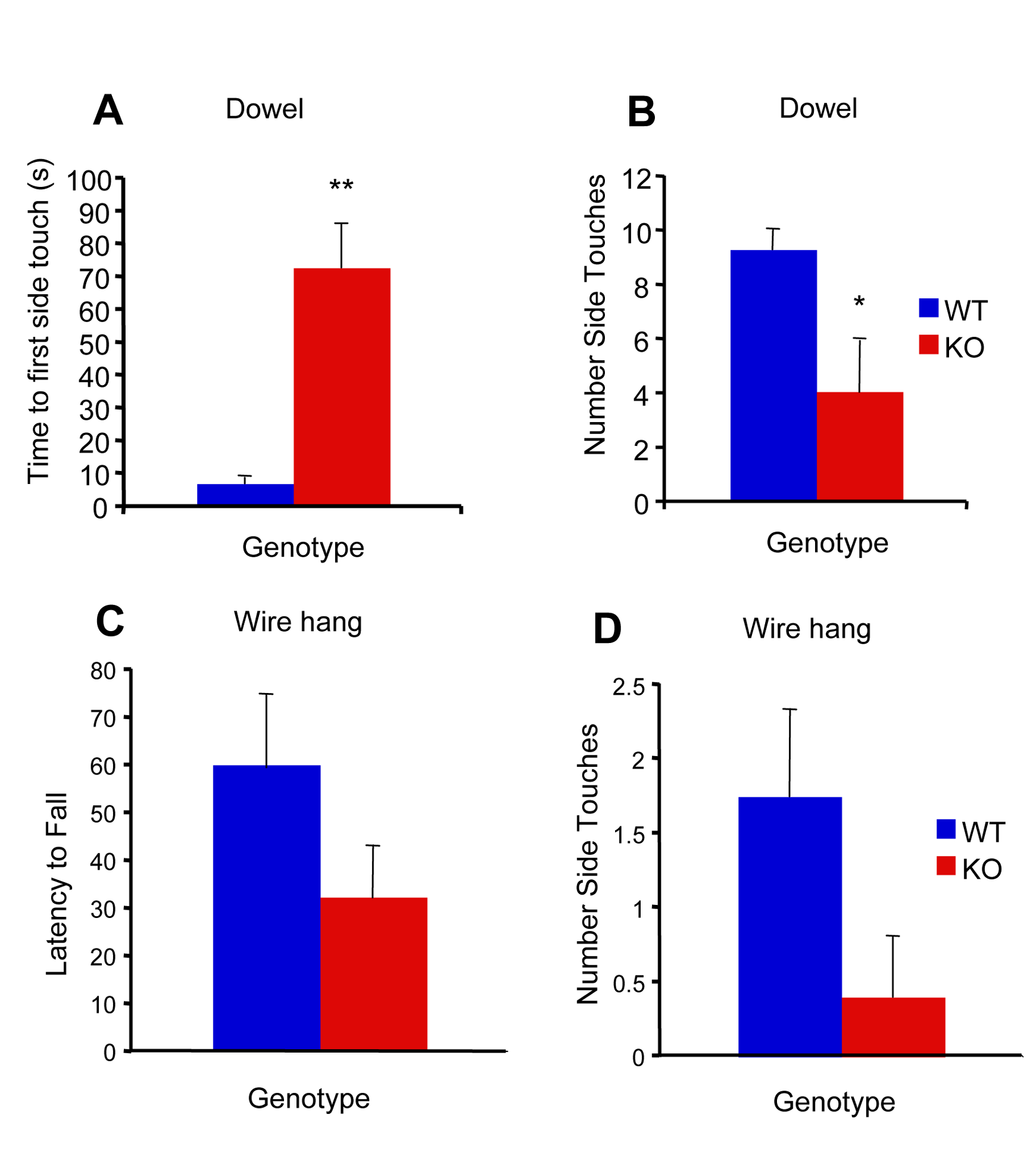

Supplement: Figure S11 — Dowel and wire hang analysis to measure gross motor ability in Atxn1−/− mice. In the Dowel test for motor coordination, mice are placed on a 0.9 cm rod and the time that it takes to reach the side and number of side touches in 2 minutes is recorded. Atxn1 −/−mice (n = 11) and wild-type littermates (n = 9) were tested at 12 weeks. (A) Atxn1 −/− mice performed poorly in the dowel, with increased latency for to reach the sides for the first time. (B) Atxn1 −/−mice also made less number of side touches in 2 min. (C and D) The wire hang test is similar to the dowel, except a wire is used instead of a rod. Atxn1 −/− mice had a trend to reach the sides fewer times in 2 minutes than the controls (D), albeit not significant (p<0.06) Error bars +/− SEM, *p<0.05,**p<0.01. (0.52 MB TIF) [file pgen.1001021.s011.tif]
