# Supplementary material for: Partial Loss of Ataxin-1 Function Contributes to Transcriptional Dysregulation in Spinocerebellar Ataxia Type 1 Pathogenesis
Source: PLoS Genet. 2010 Jul 8;6(7):e1001021. doi: 10.1371/journal.pgen.1001021 (PMC2900305; doi:10.1371/journal.pgen.1001021)
Supplement: Table S1 — List of 197 commonly altered genes in Atxn1−/− and Atxn1154Q/+ cerebella. (p<0.01 Fold Change |+/−0.1| log2 scale). (0.37 MB DOC) [file pgen.1001021.s012.doc]

**Supplementary Table S1.** List of 197 commonly altered genes in *Atxn1*^-/-^ and *Atxn1^154Q^*^/+^ cerebella (p<0.01, Fold Change |+/-0.1| log_2_ scale)

| Gene Symbol | Entrez | KI Fold-change | KO Fold-change | Adjusted P-value KI v WT | Adjusted P-value KO v WT |
| --- | --- | --- | --- | --- | --- |
| *Ttr* | 22139 | -1.459 | -0.325 | 0 | 4.00E-04 |
| *Igfbp5* | 16011 | -0.857 | -0.28 | 0 | 0 |
| *Car9* | 230099 | -0.745 | -0.453 | 0 | 1.00E-04 |
| *Prl2c4* | 26421 | -0.670 | -0.851 | 1.00E-04 | 0 |
| *Cbln2* | 12405 | -0.623 | 1.032 | 0 | 0 |
| *Kl* | 16591 | -0.601 | -0.837 | 0 | 0 |
| *Ndst4* | 64580 | -0.598 | 1.118 | 0 | 0 |
| *Tcf7l2* | 21416 | -0.536 | 0.423 | 0 | 0 |
| *328235* | 328235 | -0.532 | -1.149 | 2.00E-04 | 0 |
| *Enpp2* | 18606 | -0.444 | -0.382 | 0 | 0 |
| *17684* | 17536 | -0.437 | 0.781 | 0 | 0 |
| *C230078M14Rik* | 241175 | -0.434 | -0.296 | 0 | 0 |
| *Plcb3* | 18797 | -0.398 | -0.232 | 0 | 0 |
| *Dgkh* | 380921 | -0.391 | -0.137 | 0 | 0 |
| *Necab3* | 56846 | -0.385 | 0.303 | 0 | 0 |
| *Lhx9* | 16876 | -0.376 | 0.632 | 2.00E-04 | 6.00E-04 |
| *AW551984* | 244810 | -0.365 | 1.018 | 0 | 0 |
| *2010015L04Rik* | 544678 | -0.356 | 0.167 | 0 | 0.0079 |
| *Nrsn1* | 22360 | -0.350 | 0.259 | 0.0011 | 0.0012 |
| *1200003I10Rik* | 71719 | -0.347 | -0.213 | 0 | 1.00E-04 |
| *Cep76* | 225659 | -0.335 | -0.303 | 0 | 0 |
| *A130040M12Rik* | 319269 | -0.327 | -0.26 | 0 | 0 |
| *Kcnip4* | 80334 | -0.315 | -0.15 | 0 | 0 |
| *Nab2* | 17937 | -0.313 | -0.292 | 0 | 0 |
| *8430415E04Rik* | 74521 | -0.310 | -0.148 | 0 | 0 |
| *Prkg1* | 19091 | -0.308 | -0.15 | 0 | 3.00E-04 |
| *6330514A18Rik* | 216166 | -0.302 | -0.143 | 0 | 0.0019 |
| *Syne2* | 319565 | -0.297 | -0.329 | 0 | 0 |
| *Trpc3* | 22065 | -0.296 | -0.205 | 0 | 3.00E-04 |
| *Ldb2* | 16826 | -0.290 | 0.514 | 0.0013 | 0 |
| *Spag5* | 54141 | -0.289 | -0.169 | 0 | 0.0019 |
| *Lbp* | 16803 | -0.282 | -0.423 | 0.0091 | 0 |
| *Rasgrf2* | 19418 | -0.271 | 0.48 | 1.00E-04 | 0 |
| *Slc16a11* | 216867 | -0.268 | -0.144 | 1.00E-04 | 0.0072 |
| *Wdr76* | 241627 | -0.255 | -0.262 | 2.00E-04 | 2.00E-04 |
| *Eomes* | 13813 | -0.249 | -0.298 | 1.00E-04 | 1.00E-04 |
| *Kcnc2* | 268345 | -0.248 | 0.513 | 0.0032 | 0 |
| Gene Symbol | Entrez | KI Fold-change | KO Fold-change | Adjusted P-value KI v WT | Adjusted P-value KO v WT |
| *Sema7a* | 20361 | -0.247 | -0.171 | 0 | 0 |
| *D930020B18Rik* | 216393 | -0.244 | 0.426 | 3.00E-04 | 0 |
| *Prmt8* | 381813 | -0.243 | -0.162 | 9.00E-04 | 0 |
| *Chordc1* | 66917 | -0.242 | -0.205 | 9.00E-04 | 2.00E-04 |
| *Rmst* | 110333 | -0.237 | 0.193 | 0 | 0.0068 |
| *Zfp62* | 22720 | -0.237 | 0.159 | 0.0017 | 0.0023 |
| *Rasal1* | 19415 | -0.235 | -0.27 | 0 | 0 |
| *D7Ertd715e* | 52480 | -0.232 | 0.15 | 0 | 0.0052 |
| *LOC674800* | 674800 | -0.228 | 0.152 | 0 | 0 |
| *Nell1* | 338352 | -0.225 | -0.388 | 0 | 0 |
| *Arhgap20* | 244867 | -0.223 | -0.153 | 0 | 2.00E-04 |
| *Tuba8* | 53857 | -0.223 | -0.246 | 0.0048 | 0.0021 |
| *1200016E24Rik* | 319202 | -0.220 | -0.39 | 0 | 0 |
| *Nt5dc3* | 103466 | -0.218 | -0.27 | 0 | 0 |
| *Slc20a1* | 20515 | -0.217 | -0.13 | 0 | 0 |
| *Kcnma1* | 16531 | -0.216 | -0.199 | 0 | 0 |
| *Adamts10* | 224697 | -0.212 | 0.219 | 0 | 0 |
| *Cpeb1* | 12877 | -0.211 | -0.199 | 4.00E-04 | 0 |
| *Tbc1d8b* | 245638 | -0.208 | -0.207 | 0.0015 | 1.00E-04 |
| *Dzip3* | 224170 | -0.205 | 0.182 | 0 | 0 |
| *Slc9a3* | 105243 | -0.204 | -0.24 | 0.0018 | 1.00E-04 |
| *D6Wsu116e* | 28006 | -0.200 | -0.158 | 0 | 0 |
| *Cyp27a1* | 104086 | -0.199 | -0.2 | 0.0057 | 0 |
| *Gria3* | 53623 | -0.197 | -0.119 | 0 | 3.00E-04 |
| *Kcnq2* | 16536 | -0.197 | -0.163 | 0 | 1.00E-04 |
| *Stac* | 20840 | -0.196 | -0.205 | 0.0041 | 0 |
| *Ngef* | 53972 | -0.195 | 0.253 | 0.0017 | 4.00E-04 |
| *Pdia3* | 14827 | -0.193 | -0.176 | 1.00E-04 | 0 |
| *L1Md-Tf30* | 16736 | -0.192 | 0.214 | 0 | 0 |
| *Ak3l1* | 11639 | -0.190 | -0.189 | 6.00E-04 | 1.00E-04 |
| *Psd2* | 74002 | -0.188 | -0.185 | 0 | 0 |
| *D330017J20Rik* | 320609 | -0.188 | -0.228 | 0 | 0 |
| *Ccdc104* | 216618 | -0.187 | 0.231 | 0.0079 | 0 |
| *Cgnl1* | 68178 | -0.185 | 0.152 | 0 | 0.0052 |
| *Cast* | 12380 | -0.184 | -0.159 | 0.0055 | 0.0035 |
| *Ints8* | 72656 | -0.184 | -0.2 | 2.00E-04 | 0 |
| *Ift80* | 68259 | -0.178 | -0.143 | 3.00E-04 | 5.00E-04 |
| *Svep1* | 64817 | -0.177 | 0.156 | 0 | 0 |
| *Dusp1* | 19252 | -0.174 | -0.161 | 1.00E-04 | 0.0016 |
| *Pib5pa* | 170835 | -0.168 | -0.105 | 0 | 1.00E-04 |
| *Clmn* | 94040 | -0.167 | -0.125 | 0 | 0 |
| Gene Symbol | Entrez | KI Fold-change | KO Fold-change | Adjusted P-value KI v WT | Adjusted P-value KO v WT |
| *4732471D19Rik* | 319719 | -0.166 | -0.24 | 0.0027 | 0 |
| *Daam2* | 76441 | -0.164 | -0.13 | 0 | 7.00E-04 |
| *Grik1* | 14805 | -0.162 | -0.182 | 0.0021 | 0.0014 |
| *Gsg1l* | 269994 | -0.162 | -0.212 | 0.0064 | 0.001 |
| *Heg1* | 77446 | -0.160 | -0.143 | 5.00E-04 | 8.00E-04 |
| *Ace* | 11421 | -0.160 | -0.306 | 0.0024 | 0 |
| *Ptprm* | 19274 | -0.160 | -0.312 | 0 | 0 |
| *Gldc* | 104174 | -0.159 | -0.137 | 0 | 4.00E-04 |
| *Inpp5a* | 212111 | -0.158 | -0.121 | 0 | 0 |
| *Plxdc1* | 72324 | -0.158 | -0.307 | 0 | 0 |
| *Plcb4* | 18798 | -0.156 | -0.133 | 0 | 0 |
| *Calb1* | 12307 | -0.154 | -0.138 | 0 | 0 |
| *Dmd* | 13405 | -0.152 | 0.177 | 0 | 0 |
| *Ipo5* | 70572 | -0.150 | -0.155 | 0 | 0 |
| *Atm* | 11920 | -0.150 | -0.166 | 0 | 0 |
| *Tfrc* | 22042 | -0.149 | 0.253 | 0.0011 | 0 |
| *Mmp17* | 23948 | -0.148 | -0.166 | 0.0085 | 6.00E-04 |
| *Abca8b* | 27404 | -0.138 | -0.141 | 1.00E-04 | 0 |
| *Icmt* | 57295 | -0.138 | -0.194 | 2.00E-04 | 2.00E-04 |
| *Ptpru* | 19273 | -0.137 | -0.149 | 0 | 0 |
| *Kif5b* | 16573 | -0.133 | 0.154 | 0 | 0 |
| *Ints4* | 101861 | -0.131 | -0.124 | 0.0058 | 0.001 |
| *Ryr3* | 20192 | -0.130 | -0.294 | 0 | 0 |
| *Homer3* | 26558 | -0.126 | -0.191 | 0.0033 | 0 |
| *Hspa4l* | 18415 | -0.125 | -0.129 | 0 | 0 |
| *Serinc3* | 26943 | -0.125 | -0.145 | 1.00E-04 | 1.00E-04 |
| *Pla2g7* | 27226 | -0.120 | -0.127 | 0.004 | 2.00E-04 |
| *Ptpn4* | 19258 | -0.118 | 0.119 | 1.00E-04 | 0 |
| *Adam23* | 23792 | -0.118 | -0.147 | 0 | 0 |
| *Ankle2* | 71782 | -0.117 | -0.104 | 0.0037 | 0.0024 |
| *Mtr* | 238505 | -0.112 | -0.104 | 0.0026 | 0.0037 |
| *Cap2* | 67252 | -0.111 | -0.224 | 0.0017 | 0 |
| *Med14* | 26896 | -0.110 | -0.141 | 1.00E-04 | 0 |
| *Megf11* | 214058 | -0.109 | -0.19 | 0.0012 | 0 |
| *2700050L05Rik* | 214764 | -0.106 | -0.171 | 0.0083 | 0 |
| *Atp2a3* | 53313 | -0.106 | -0.232 | 0 | 0 |
| *Slc12a2* | 20496 | -0.105 | -0.148 | 1.00E-04 | 0 |
| *Itpr1* | 16438 | -0.102 | -0.157 | 0 | 0 |
| *Col18a1* | 12822 | -0.102 | -0.176 | 0.0027 | 0 |
| *Ckap5* | 75786 | -0.101 | -0.114 | 0 | 0 |
| *Ltbp4* | 108075 | 0.102 | -0.147 | 0.0073 | 1.00E-04 |
| Gene Symbol | Entrez | KI Fold-change | KO Fold-change | Adjusted P-value KI v WT | Adjusted P-value KO v WT |
| *Dync1i1* | 13426 | 0.105 | -0.16 | 8.00E-04 | 0 |
| *Spnb1* | 20741 | 0.106 | 0.102 | 0 | 0 |
| *Dab1* | 13131 | 0.108 | 0.178 | 0.0097 | 0 |
| *Nos1* | 18125 | 0.110 | -0.264 | 0 | 0 |
| *Ap2a1* | 11771 | 0.112 | -0.125 | 0 | 0 |
| *Mag* | 17136 | 0.113 | -0.118 | 0.0041 | 0 |
| *Men1* | 17283 | 0.114 | -0.151 | 0.0092 | 0.0014 |
| *BC057079* | 230393 | 0.117 | 0.118 | 4.00E-04 | 0.0014 |
| *Kif13a* | 16553 | 0.118 | 0.366 | 0 | 0 |
| *Atp6v1f* | 66144 | 0.122 | -0.132 | 0.0047 | 0.0073 |
| *Cacna1c* | 12288 | 0.123 | -0.122 | 0 | 0 |
| *Col9a3* | 12841 | 0.125 | -0.123 | 0.0013 | 0.0039 |
| *Phf2* | 18676 | 0.126 | -0.108 | 0.0012 | 0.0062 |
| *Crtc1* | 382056 | 0.128 | -0.119 | 0 | 0 |
| *Ap3b2* | 11775 | 0.134 | -0.107 | 0 | 1.00E-04 |
| *Man2b2* | 17160 | 0.134 | -0.234 | 0.001 | 0 |
| *Usp11* | 236733 | 0.137 | -0.153 | 0.0019 | 0 |
| *Stard10* | 56018 | 0.140 | 0.113 | 4.00E-04 | 0.007 |
| *BC037034* | 231807 | 0.140 | -0.112 | 0.0016 | 0.0099 |
| *Rtn1* | 104001 | 0.140 | -0.129 | 0 | 1.00E-04 |
| *9030409G11Rik* | 71529 | 0.144 | -0.198 | 0.0058 | 0 |
| *Rims3* | 242662 | 0.146 | 0.142 | 5.00E-04 | 0 |
| *Tulp4* | 68842 | 0.147 | 0.188 | 0 | 0 |
| *Meg3* | 17263 | 0.151 | 0.119 | 0 | 3.00E-04 |
| *6430550H21Rik* | 245386 | 0.152 | -0.16 | 6.00E-04 | 4.00E-04 |
| *Cxx1a* | 66158 | 0.153 | -0.115 | 0 | 1.00E-04 |
| *Vtn* | 22370 | 0.154 | -0.149 | 6.00E-04 | 1.00E-04 |
| *Trak1* | 67095 | 0.155 | 0.108 | 0 | 0.006 |
| *Tle3* | 21887 | 0.156 | -0.175 | 6.00E-04 | 0 |
| *Peli2* | 93834 | 0.157 | 0.181 | 0.0027 | 5.00E-04 |
| *Slc6a15* | 103098 | 0.158 | 0.217 | 1.00E-04 | 0 |
| *Ube2o* | 217342 | 0.159 | -0.119 | 0 | 0.0014 |
| *NA* | NA | 0.160 | 0.102 | 4.00E-04 | 0.0055 |
| *Otud7a* | 170711 | 0.162 | 0.174 | 0 | 0 |
| *Syngr3* | 20974 | 0.166 | -0.151 | 1.00E-04 | 0.0042 |
| *Ppp2r2b* | 72930 | 0.178 | 0.143 | 0.0035 | 0.0032 |
| *Tnrc4* | 78784 | 0.182 | 0.138 | 0 | 7.00E-04 |
| *Prdm8* | 77630 | 0.183 | -0.178 | 0 | 0 |
| *Cryl1* | 68631 | 0.184 | -0.219 | 0.0046 | 0.0053 |
| *Trim67* | 330863 | 0.192 | 0.338 | 0 | 0 |
| *Lrrk2* | 66725 | 0.194 | -0.144 | 0 | 0 |
| Gene Symbol | Entrez | KI Fold-change | KO Fold-change | Adjusted P-value KI v WT | Adjusted P-value KO v WT |
| *Tiam2* | 24001 | 0.199 | 0.52 | 0.0031 | 0 |
| *Etv5* | 104156 | 0.200 | 0.164 | 0 | 0 |
| *Acvr1c* | 269275 | 0.201 | 0.212 | 0 | 0 |
| *Zic3* | 22773 | 0.202 | -0.212 | 0.0013 | 0.0023 |
| *Plxdc2* | 67448 | 0.207 | 0.139 | 0 | 0 |
| *Tgfb3* | 21809 | 0.208 | 0.222 | 0.0027 | 7.00E-04 |
| *Adcyap1r1* | 11517 | 0.209 | 0.237 | 0 | 0 |
| *Sez6* | 20370 | 0.220 | -0.137 | 0 | 1.00E-04 |
| *Slc25a18* | 71803 | 0.221 | 0.241 | 1.00E-04 | 0 |
| *Dpysl3* | 22240 | 0.221 | 0.184 | 0.0018 | 0.0059 |
| *Epha7* | 13841 | 0.226 | -0.231 | 0 | 0 |
| *6430517E21Rik* | 240843 | 0.231 | 0.18 | 2.00E-04 | 0.0018 |
| *Adamts1* | 11504 | 0.231 | -0.22 | 0 | 1.00E-04 |
| *Maml3* | 433586 | 0.232 | 0.214 | 0.0018 | 0.0069 |
| *Ebf1* | 13591 | 0.249 | -0.123 | 0 | 0.0075 |
| *Nrip2* | 60345 | 0.257 | 0.187 | 1.00E-04 | 2.00E-04 |
| *Cpne4* | 74020 | 0.263 | 0.616 | 0.0026 | 0 |
| *Creb5* | 231991 | 0.268 | 0.549 | 0.0046 | 0.0016 |
| *Brunol4* | 108013 | 0.270 | 0.164 | 0 | 0 |
| *Dusp26* | 66959 | 0.284 | 0.216 | 0 | 8.00E-04 |
| *Lgr5* | 14160 | 0.285 | -0.196 | 0 | 0 |
| *NA* | NA | 0.297 | 0.194 | 4.00E-04 | 0 |
| *9430031J16Rik* | 241134 | 0.299 | 0.267 | 0 | 1.00E-04 |
| *Adamts18* | 208936 | 0.301 | 0.194 | 0 | 0 |
| *Sipa1l2* | 244668 | 0.303 | 0.255 | 0 | 0 |
| *Syn3* | 27204 | 0.317 | 0.211 | 0 | 0 |
| *Ccnd1* | 12443 | 0.322 | 0.25 | 4.00E-04 | 0.006 |
| *Hist1h3d* | 319149 | 0.341 | -0.15 | 0 | 1.00E-04 |
| *Aloxe3* | 23801 | 0.395 | 0.225 | 0 | 3.00E-04 |
| *Robo1* | 19876 | 0.398 | 0.418 | 0 | 0 |
| *Rgs17* | 56533 | 0.417 | 0.235 | 0 | 0 |
| *Dusp4* | 319520 | 0.466 | 0.369 | 1.00E-04 | 0 |
| *Angpt1* | 11600 | 0.477 | 0.195 | 0 | 0.005 |
| *NA* | NA | 0.560 | 0.893 | 2.00E-04 | 0 |
| *AY036118* | 170798 | 0.577 | -0.359 | 0 | 0 |
| *Synpr* | 72003 | 0.597 | 0.197 | 0 | 0.0078 |
| *Eif2s3y* | 26908 | 1.275 | 0.171 | 0 | 5.00E-04 |
