# Supplementary material for: Partial Loss of Ataxin-1 Function Contributes to Transcriptional Dysregulation in Spinocerebellar Ataxia Type 1 Pathogenesis
Source: PLoS Genet. 2010 Jul 8;6(7):e1001021. doi: 10.1371/journal.pgen.1001021 (PMC2900305; doi:10.1371/journal.pgen.1001021)
Supplement: Table S2 — KEGG pathway analysis of the 197 commonly altered genes in Atxn1 −/− and Atxn1154Q /+ cerebella. (0.06 MB DOC) [file pgen.1001021.s013.doc]

**Supplementary Table S2.** KEGG pathway analysis of the 197 commonly altered genes in *Atxn1*^-/-^ and *Atxn1^154Q^*^/+^ cerebella

| Term | PValue | Genes down-regulated in both KO and KI | Fold Enrichment |
| --- | --- | --- | --- |
| mmu04070:  Phosphatidylinositol signaling system | 2.72E-04 | PLCB3,PLCB4,DGKZ,DGKH,  INPP4A,INPP5A,ITPR1 | 7.53 |
| mmu04730:  Long-term depression | 3.40E-04 | CRHR1,PLCB3,PLCB4,GRID2,GRIA3,PRKG1,ITPR1 | 7.23 |
| mmu04540:  Gap junction | 5.40E-03 | TUBA8,ADCY1,PLCB3,PLCB4,PRKG1,ITPR1 | 5.18 |
| mmu04020:  Calcium signaling pathway | 9.72E-03 | ATP2B2,ADCY1,PLCB3,PLCB4ATP2A2,ATP2A3,RYR3,ITPR1 | 3.27 |
| mmu02010:  ABC transporters | 1.63E-02 | ABCA8B,ABCA2,ABCA1,  ABCC8 | 7.3 |
| mmu00562:  Inositol phosphate metabolism | 2.43E-02 | PLCB3,PLCB4,INPP4A,INPP5A | 6.28 |
| mmu05010:  Alzheimer's disease | 2.76E-02 | SDHA,PLCB3,PLCB4,ATP2A2,ATP2A3,RYR3,ITPR1 | 2.97 |
|  |  |  |  |
| Term | PValue | Genes up-regulated in both KO and KI | Fold Enrichment |
| mmu05212:  Pancreatic cancer | 2.53E-02 | CCND1,TGFB3,ACVR1C | 11.36 |
| mmu05220:  Chronic myeloid leukemia | 2.79E-02 | CCND1,TGFB3,ACVR1C | 10.76 |
|  |  |  |  |
| Term | PValue | Genes changes in opposite direction between KO and KI | Fold Enrichment |
| mmu00020:  Citrate cycle (TCA cycle) | 3.05E-02 | PCX,ACLY,MDH2 | 10.67 |
| mmu04120:  Ubiquitin mediated proteolysis | 3.09E-02 | ANAPC2,UBE2O,UBE3B,  WWP2,MID1 | 4.08 |
